# Global Disparities of Cancer and Its Projected Burden in 2050

**DOI:** 10.1001/jamanetworkopen.2024.43198

**Published:** 2024-11-05

**Authors:** Habtamu Mellie Bizuayehu, Kedir Y. Ahmed, Getiye Dejenu Kibret, Abel F. Dadi, Sewunet Admasu Belachew, Tanmay Bagade, Teketo Kassaw Tegegne, Rebecca L. Venchiarutti, Kelemu Tilahun Kibret, Aklilu Habte Hailegebireal, Yibeltal Assefa, Md Nuruzzaman Khan, Amanuel Abajobir, Kefyalew Addis Alene, Zelalem Mengesha, Daniel Erku, Daniel A. Enquobahrie, Tsion Zewdu Minas, Eyaya Misgan, Allen G. Ross

**Affiliations:** 1 Rural Health Research Institute, Charles Sturt University, Orange, Australia; 2 School of Public Health, Faculty of Health, University of Technology Sydney, Ultimo, Australia; 3 Centre for Health Systems and Safety Research, Australian Institute of Health Innovation, Faculty of Medicine, Health, and Human Sciences, Macquarie University, Sydney, Australia; 4 Menzies School of Health Research, Charles Darwin University, Darwin, Northern Territory, Australia; 5 Addis Continental Institute of Public Health, Addis Ababa, Ethiopia; 6 School of Public Health, Faculty of Medicine, The University of Queensland, Brisbane, Australia; 7 Center for Women’s Health Research, College of Health, Medicine, and Wellbeing, The University of Newcastle, Callaghan, Australia; 8 Institute for Physical Activity and Nutrition, Deakin University, Geelong, Australia; 9 Sydney School of Public Health, Faculty of Medicine and Health, The University of Sydney, Camperdown, Australia; 10 Department of Head and Neck Surgery, Chris O’Brien Lifehouse, Sydney, Australia; 11 Global Centre for Preventive Health and Nutrition, Institute for Health Transformation, Faculty of Health, Deakin University, Geelong, Australia; 12 School of Public Health, College of Medicine and Health Sciences, Wachemo University, Hosanna, Ethiopia; 13 Department of Population Science, Jatiya Kabi Kazi Nazrul Islam University, Mymensingh, Bangladesh; 14 Nossal Institute for Global Health, Melbourne School of Population and Global Health, The University of Melbourne, Melbourne, Australia; 15 Sexual, Reproductive, Maternal, Newborn, Child and Adolescent Health Unit, Health and Wellbeing Theme, African Population and Health Research Center, Nairobi, Kenya; 16 School of Population Health, Faculty of Health Sciences, Curtin University, Perth, Australia; 17 Geospatial and Tuberculosis Research Team, Telethon Kids Institute, Perth, Australia; 18 Health Research Institute, Faculty of Health, University of Canberra, Canberra, Australia; 19 Health Economics and Financing Practice, Global Health Systems Innovation Group, Management Sciences for Health, Arlington, Virginia; 20 Department of Epidemiology, School of Public Health, University of Washington, Seattle; 21 Department of Pathology, School of Medicine, Johns Hopkins University, Baltimore, Maryland; 22 Laboratory of Human Carcinogenesis, Center for Cancer Research, National Cancer Institute, Bethesda, Maryland; 23 Center for Innovative Drug Development and Therapeutic Trials for Africa, Addis Ababa University, Addis Ababa, Ethiopia; 24 Department of Gynecology and Obstetrics, College of Medicine and Health Sciences, Bahir Dar University, Bahir Dar, Ethiopia; 25 University of Rwanda, College of Medicine and Health Sciences, Kigali, Rwanda

## Abstract

**Importance:**

Cancer prevention and care efforts have been challenged by the COVID-19 pandemic and armed conflicts, resulting in a decline in the global Human Development Index (HDI), particularly in low- and middle-income countries. These challenges and subsequent shifts in health care priorities underscore the need to continuously monitor cancer outcome disparities and statistics globally to ensure delivery of equitable and optimal cancer prevention and care in uncertain times.

**Objective:**

To measure the global burden of 36 cancers in 2022 by sex, age, and geographic location and to project future trends by 2050.

**Design, Setting, and Participants:**

This cross-sectional study used population-based data from 2022 in 185 countries and territories were obtained from the Global Cancer Observatory database. Data extraction and analysis were carried out in April 2024.

**Main Outcomes and Measures:**

Counts, rates, prevalence, mortality to incidence ratios (MIRs), and demography-based projections were used to characterize current and future cancer burden.

**Results:**

This population-based study included 36 cancer types from 185 countries and territories. By 2050, 35.3 million cancer cases worldwide are expected, a 76.6% increase from the 2022 estimate of 20 million. Similarly, 18.5 million cancer deaths are projected by 2050, an 89.7% increase from the 2022 estimate of 9.7 million. Cancer cases and deaths are projected to nearly triple in low-HDI countries by 2050, compared to a moderate increase in very high–HDI countries (142.1% vs 41.7% for cancer cases and 146.1% vs 56.8% for cancer deaths). Males had a higher incidence and greater number of deaths in 2022 than females, with this disparity projected to widen by up to 16.0% in 2050. In 2022, the MIR for all cancers was 46.6%, with higher MIRs observed for pancreatic cancer (89.4%), among males (51.7%), among those aged 75 years or older (64.3%), in low-HDI countries (69.9%), and in the African region (67.2%).

**Conclusions and Relevance:**

In this cross-sectional study based on data from 2022, cancer disparities were evident across HDI, geographic regions, age, and sex, with further widening projected by 2050. These findings suggest that strengthening access to and quality of health care, including universal health insurance coverage, is key to providing evidence-based cancer prevention, diagnostics, and care.

## Key Points

**Question:**

What were the global disparities in cancer burden by cancer type, sex, age, Human Development Index (HDI), regions, and countries and territories in 2022, and how are these epidemiological patterns projected to change by 2050?

**Findings:**

In this cross-sectional study of data for 36 cancer types from 185 countries and territories, cancer disparities were evident across HDI, region, age, and sex in 2022 and are projected to widen by 2050. Cancer cases and deaths are expected to rise by 77% and 90% in 2050, respectively, with a 3-fold increase in low-HDI countries compared with a modest increase in very high–HDI countries (142% vs 42% for cancer cases and 146% vs 57% for cancer deaths).

**Meaning:**

These findings suggest that enhancing health care systems for cancer prevention, early diagnosis, management, and treatment is vital to better address existing disparities in cancer outcomes and slow projected trends.

## Teaser

This study uses population-based data from the Global Cancer Observatory (GLOBOCAN) to assess the global burden of 36 cancers in 2022 by sex, age, and geographic location and to project future trends by 2050.

## Introduction

Global cancer prevention and care efforts underwent major disruptions after 2020, driven by the ongoing effects of the COVID-19 pandemic and further exacerbated by persistent armed conflicts, changing health care funding priorities, and a cost-of-living crisis.^1,2^ Between 2020 and 2022, the global Human Development Index (HDI), a composite measure of critical human development indicators such as life expectancy, education, and gross national income per capita, declined substantially for 2 consecutive years.^1^ The consequences of these ongoing disruptions may disproportionately affect cancer care in certain regions and for individuals based on certain sociodemographic characteristics such as sex and age, resulting in disparities that can be assessed through the mortality to incidence ratio (MIR).^3,4,5,6,7^ The MIR, for which higher values indicate higher case fatality and poor survival, has been used in the literature^3,4,5,6,7^ and in government reports^8,9^ in Australia^9^ and the US^8^ to assess cancer outcome equity.

Previous studies focused on the MIR were conducted using data collected prior to 2020^3,4,5,6,7^ and data on selected cancer types, such as lung,^5^ liver,^6^ and gastric cancer.^3,4^ As already noted, global disruptions and shifts in health priorities^1,2^ underscore the need to continuously monitor cancer statistics globally to ensure delivery of equitable and optimal cancer prevention and care in uncertain times. To support evidence-based decision-making with respect to health care resource allocation, we sought to analyze the MIR for 36 cancers and to assess disparities by geographic region, sex, and age using the latest Global Cancer Observatory (GLOBOCAN) data released in 2024.^10,11^ In addition, this study analyzed cancer rates, prevalence, and projections for 2050 by age, sex, and region, providing a more comprehensive assessment of global cancer burden.

## Methods

### Data Sources

This cross-sectional study used population-based cancer data from GLOBOCAN 2022, curated by the International Agency for Research on Cancer (IARC).^11,12,13^ The GLOBOCAN repository aggregates publicly available global cancer-related data, encompassing data from each country or territory. At the national level, GLOBOCAN estimates cancer cases, deaths, rates, and prevalence using population-based administrative data sources such as cancer registries, civil and vital statistics registration systems, or modeling, applying robust methodologies tailored to the specific context of each country or territory.^11,12,14,15^ This study followed the relevant portions of the Strengthening the Reporting of Observational Studies in Epidemiology (STROBE) reporting guideline. As the GLOBOCAN project solely uses publicly accessible and secondary data, the IARC Ethics Committee deemed that ethical approval was not required for this study.

### Measures

This study included all cancer types available in the GLOBOCAN database, totaling 36 types. Specific cancer types were identified by referencing *International Statistical Classification of Diseases, Tenth Revision* diagnosis codes (eTable 1 in Supplement 1).^12,16^ The dataset was further stratified by sex, age, country or territory, region, and HDI.^1,12,16^ Age was grouped as 0 to 19, 20 to 39, 40 to 64, 65 to 74, and 75 years or older based on importance for epidemiology, policy, public health, and clinical practice.^17,18^ In line with the 2022 United Nations Development Programme classification, HDI was reported in 4 tiers (ie, low, medium, high, and very high).^1^ Countries and territories were assigned to 1 of 6 regions: Africa, Asia, Europe, Latin America and the Caribbean, North America, and Oceania.

### Statistical Analysis

In this study, we report various measures of burden, including counts, rates, prevalence, and MIR. Incidence and mortality rates were determined by dividing the number of cases and deaths, respectively, in 2022 by the total population in the same year, with age-standardized incidence rates (ASIRs) and age-standardized mortality rates (ASMRs) calculated by adjusting their crude rates with the Segi-Doll World Standard Population, as computed in 1966.^10,12,19^ To estimate the MIR, the ASMR was divided by the ASIR and multiplied by 100 to obtain a percentage, with a higher MIR indicating poorer survival after a cancer diagnosis.^4,20,21^ Cancer prevalence was calculated by dividing the number of persons diagnosed with cancer and known to be alive in a specific period by the total population during that period.^10,11^ To project future cancer cases and deaths, demographic projections were used, assuming that the 2022 cancer rates remain stable.^13,22,23^ Hence, the 2050 cancer estimates were generated by applying the 2022 standardized rates to the 2050 population predicted by the United Nations Development Programme.^1^ Further methodological details are provided in the eMethods in Supplement 1.

Data analysis was carried out using R, version 4.3.1 (R Project for Statistical Computing), Excel (Microsoft Corp), and the GLOBOCAN online tabulation and visualization tools (IARC). Data extraction and analysis was carried out in April 2024.

## Results

### Cancer Counts, Rate, Prevalence, and Projections

This study assessed cancer burden disparity and projections for 36 cancers based on population-based data from 185 countries and territories. By 2050, the total number of cancer cases is projected to increase to 35.3 million, an increase of 76.6% from the 2022 estimate of 20 million (Table 1). Similarly, cancer deaths are estimated to reach 18.5 million, an increase of 89.7% from the 2022 estimate of 9.7 million. In 2022, the prevalence of cancer was 178.9 cases per 100 000 persons (Table 1). The 5-year period prevalence (2018-2022) was 678.6 cases per 100 000 persons (eTable 2 in Supplement 1). Breast cancer was the most prevalent cancer, accounting for 13.3% of patients with cancer who were diagnosed and alive in 2022. The next most prevalent cancers were prostate, colorectum, lung, and nonmelanoma skin cancer (eTable 3 in Supplement 1). In 2022, lung cancer was the leading incident cancer (accounting for 12.4% of new cases) and cancer deaths (accounting for 18.7% of cancer deaths). In 2050, lung cancer is projected to be the leading cause of cancer (accounting for 13.1% of new cases) and cancer deaths (accounting for 19.2% of cancer deaths). The top 10 most prevalent cancers are presented in eTable 3 in Supplement 1.

**Table 1.** Prevalence and MIR of Cancer in 2022 and Projection of Cancer Cases and Deaths in 2050 by Cancer Type

Cancer type	Females						Males						Both sexes					
	Prevalence in 2022^a^	MIR in 2022	No. of cases in 2050	Change, %	No. of deaths in 2050	Change, %	Prevalence in 2022^a^	MIR in 2022	No. of cases in 2050	Change, %	No. of deaths in 2050	Change, %	Prevalence in 2022^a^	MIR in 2022	No. of cases in 2050	Change, %	No. of deaths in 2050	Change, %
All cancers	179.0	41.3	16 280 527	68.5	7 989 377	85.2	178.8	51.7	19 000 529	84.3	10 490 923	93.2	178.9	46.6	35 281 056	76.6	18 480 300	89.7
All cancers, excluding NMSC	168.0	42.7	15 232 802	66.0	7 924 506	85.0	162.6	54.9	17 350 353	81.4	10 405 366	93.0	165.3	48.9	32 583 155	73.9	18 329 871	89.5
Lip, oral cavity	2.2	47.8	208 183	72.3	102 329	77.6	4.7	48.3	433 380	61.1	213 557	63.3	3.5	47.5	641 563	64.6	315 886	67.6
Salivary glands	0.47	36.7	38 527	59.7	17 802	78.9	0.59	43.9	52 858	70.7	25 750	84.1	0.53	41.1	91 385	65.9	43 552	81.9
Oropharynx	0.39	46.2	32 933	64.2	16 827	77.4	1.6	47.9	141 181	63.5	72 633	69.6	1	48.2	174 114	63.6	89 460	71.0
Nasopharynx	0.63	53.4	49 771	45.8	31 027	60.1	1.6	63.2	126 542	46.7	85 600	58.2	1.1	59.2	176 313	46.4	116 627	58.7
Hypopharynx	0.22	41.4	22 223	56.7	10 567	66.7	1.1	45.6	119 433	65.7	59 698	72.7	0.67	46.1	141 656	64.2	70 265	71.8
Esophagus	2.2	84.6	265 446	82.0	238 831	88.1	5.6	85.5	657 192	79.9	586 747	84.3	3.9	86.0	922 638	80.5	825 578	85.4
Stomach	4.8	65.0	637 677	86.8	452 907	94.7	8.9	67.2	1 178 681	87.9	832 310	94.7	6.9	66.3	1 816 357	87.5	1 285 217	94.7
Colorectum	16.8	42.8	1 593 471	85.9	826 029	104.3	20.7	45.2	1 980 340	85.2	1 009 371	102.0	18.7	44.0	3 573 811	85.5	1 835 400	103.0
Liver	3.6	85.4	487 751	83.7	447 916	89.1	8.2	85.8	1 036 750	72.6	923 551	77.0	5.9	86.1	1 524 501	76.0	1 371 467	80.8
Gallbladder	1.2	71.4	144 782	83.4	107 617	86.7	0.64	71.6	83 878	92.7	61 722	96.5	0.89	69.2	228 660	86.7	169 339	90.2
Pancreas	2.8	87.5	482 093	99.8	450 072	104.8	3.1	90.9	516 570	91.5	485 966	96.3	3	89.4	998 663	95.4	936 038	100.3
Larynx	0.43	51.1	39 726	69.8	23 786	83.3	3.1	54.3	286 039	72.5	163 895	81.3	1.8	52.6	325 765	72.2	187 681	81.6
Lung	12.6	60.5	1 665 164	83.3	1 146 109	96.2	19.7	77.3	2 954 902	88.0	2 401 765	94.8	16.2	71.2	4 620 066	86.2	3 547 874	95.2
Melanoma of skin	3.6	14.8	260 785	71.8	50 158	96.6	4.1	17.6	338 780	88.3	67 063	102.2	3.8	16.6	599 565	80.7	117 222	99.8
NMSC	11.0	6.0	1 047 724	113.9	64 871	118.2	16.2	5.5	1 650 176	121.6	85 557	115.6	13.6	5.7	2 697 901	118.5	150 429	116.7
Mesothelioma	0.14	81.3	17 028	84.6	14 069	93.0	0.3	83.3	44 002	105.5	38 890	115.1	0.22	78.6	61 030	99.2	52 959	108.7
Kaposi sarcoma	0.16	50.0	14 597	30.4	7135	28.8	0.37	44.6	33 537	36.2	13 908	30.9	0.27	46.3	48 134	34.4	21 043	30.1
Breast	47.9	27.1	3 553 037	54.7	1 138 155	70.9	NA	NA	NA	NA	NA	NA	47.9	27.0	3 553 037	54.7	1 138 155	70.9
Vulva	0.94	36.1	88 369	86.7	37 263	100.6	NA	NA	NA	NA	NA	NA	0.94	36.1	88 369	86.7	37 263	100.6
Vagina	0.33	41.7	31 938	69.7	14 777	79.3	NA	NA	NA	NA	NA	NA	0.33	41.7	31 938	69.7	14 777	79.3
Cervix uteri	11.9	50.4	948 116	43.2	542 825	55.6	NA	NA	NA	NA	NA	NA	11.9	50.4	948 116	43.2	542 825	55.6
Corpus uteri	9.0	20.2	676 296	60.9	183 093	87.4	NA	NA	NA	NA	NA	NA	9	20.2	676 296	60.9	183 093	87.4
Ovary	6.1	59.7	503 790	55.2	351 164	69.7	NA	NA	NA	NA	NA	NA	6.1	59.7	503 790	55.2	351 164	69.7
Penis	NA	NA	NA	NA	NA	NA	0.69	35.4	67 002	77.7	25 361	84.6	0.69	35.4	67 002	77.7	25 361	84.6
Prostate	NA	NA	NA	NA	NA	NA	30.5	24.8	2 879 501	96.2	939 534	136.4	30.5	24.8	2 879 501	96.2	939 534	136.4
Testis	NA	NA	NA	NA	NA	NA	1.6	12.4	88 362	22.7	12 697	40.0	1.6	12.4	88 362	22.7	12 697	40.0
Kidney	3.1	31.7	269 210	71.4	109 731	97.3	5.4	33.9	476 581	71.6	195 131	94.5	4.3	34.1	745 791	71.5	304 861	95.5
Bladder	2.8	33.3	282 673	97.7	121 651	121.5	9.6	33.3	946 705	100.9	372 719	125.0	6.2	32.1	1 229 377	100.1	494 370	124.1
Brain, central nervous system	2.9	71.0	232 925	57.4	180 912	66.5	3.4	79.5	270 984	56.0	228 738	63.6	3.2	74.3	503 910	56.6	409 650	64.9
Thyroid	12.6	3.9	819 021	33.2	57 356	89.5	4.1	7.6	284 794	37.9	33 360	93.5	8.3	4.8	1 103 816	34.4	90 715	91.0
Hodgkin lymphoma	0.67	23.4	46 849	39.0	14 784	63.2	0.96	28.2	67 226	37.8	21 444	56.8	0.82	25.3	114 075	38.3	36 229	59.4
Non-Hodgkin lymphoma	4.7	41.3	423 663	75.1	203 349	90.2	5.9	45.5	536 509	72.3	271 565	88.9	5.3	42.9	960 173	73.5	474 914	89.5
Multiple myeloma	1.7	61.3	154 282	83.4	106 368	95.5	2.0	61.9	194 362	87.2	133 209	98.9	1.8	61.1	348 644	85.5	239 577	97.4
Leukemia	3.8	59.1	341 267	63.2	232 413	75.9	5.1	59.7	459 432	65.2	309 616	78.7	4.5	58.5	800 698	64.3	542 029	77.5

Abbreviations: MIR, mortality to incidence ratio; NA, not applicable; NMSC, nonmelanoma skin cancer.

^a^ The number of cases diagnosed in 2022 and survived in the same year, as well as the 5-year period prevalence, is presented in eTable 2 in Supplement 1.

**Figure 1.**
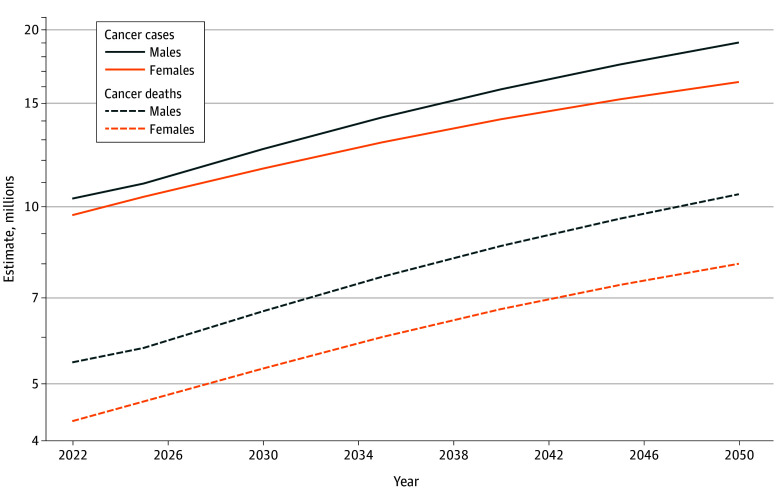
Worldwide Projected Number of Cancer Cases and Deaths by Sex, 2022-2050

Between 2022 and 2050, an upward trend in cancer cases and deaths for both males and females is expected (Figure 1). However, a slightly higher increase in cancer cases (15.8% higher) and deaths (8.0% higher) in 2050 is projected among males compared with females. Among males, 19.0 million cancer cases are anticipated in 2050, an increase of 84.3% from 10.3 million in 2022 (Table 1). The projected number of cases among females in 2050 is 16.3 million, an increase of 68.5% from 9.7 million cases in 2022. Cancer deaths among males are projected to reach 10.5 million in 2050, a 93.2% increase from the 2022 estimate of 5.4 million. The projected number of deaths for females is 8.0 million, an 85.2% increase from the 2022 estimate of 4.3 million.

The 2022 ASIR and ASMR varied by HDI, with the ASIR more than twice as high in very high–HDI countries (285.7 cases per 100 000 persons) compared with low-HDI countries (110.6 cases per 100 000 persons) (Table 2). The ASMR ranged from 73.1 deaths per 100 000 persons in medium-HDI countries to 96.0 deaths per 100 000 persons in very high–HDI countries (Table 3). In 2050, a 3-fold increase in cancer cases is projected in low-HDI countries (increase of 142.1%) compared with a projected increase of 41.7% in very high–HDI countries (Table 2). Cancer deaths in low-HDI countries are expected to increase by 146.1% compared with an increase of 56.8% in very high–HDI countries (Table 3).

**Table 2.** Distribution of Cancer Cases, Rates, and Prevalence in 2022 and Projected Cases by 2050 Across Age, HDI, and Region

Characteristic	Females					Males					Both sexes				
	No. of cases in 2022	ASIR^a^	Prevalence^b^	No. of cases in 2050	Change, %	No. of cases in 2022	ASIR^a^	Prevalence^b^	No. of cases in 2050	Change, %	No. of cases in 2022	ASIR^a^	Prevalence^b^	No. of cases in 2050	Change, %
Age group, y															
0-19	121 659	9.7	6.9	126 803	4.2	154 054	11.6	8.3	156 508	1.6	275 713	10.7	7.6	283 311	2.8
20-39	819 008	67.1	55.1	923 980	12.8	429 222	34.2	26.6	483 853	12.7	1 248 230	50.3	40.5	1 407 834	12.8
40-64	4 194 578	381.0	240.2	5 627 751	34.2	3 674 832	338.9	293.9	5 238 037	42.5	7 869 410	359.3	267.1	10 865 788	38.1
65-74	2 220 224	850.1	624.2	3 703 685	66.8	3 200 612	1369.1	962.5	5 508 950	72.1	5 420 836	1094.7	784.0	9 212 636	70.0
≥75	2 309 420	1294.7	845.1	5 898 306	112.0	2 852 890	2262	1458.3	7 613 180	166.9	5 162 310	1697.6	1101.7	13 511 487	161.7
Region															
Africa	679 184	140.7	55.6	1 592 426	134.5	506 032	125.7	40.3	1 244 910	146.0	1 185 216	132.3	48.0	2 837 336	139.4
Asia	4 732 544	157.7	141.6	7 946 498	67.9	5 093 995	174.3	133.9	9 427 687	85.1	9 826 539	164.4	137.7	17 374 185	76.8
Europe	2 112 119	253.4	437.1	2 450 585	16.0	2 359 303	319.6	504.3	3 118 658	32.2	4 471 422	280.0	469.6	5 569 243	24.6
Latin America and the Caribbean	782 217	177.4	164.3	1 365 053	74.5	768 843	199.9	158.5	1 506 210	95.9	1 551 060	186.0	161.4	2 871 263	85.1
Northern America	1 235 140	340.7	553.4	1 735 508	40.5	1 438 034	397.7	637.8	2 243 908	56.0	2 673 174	364.7	595.2	3 979 416	48.9
Oceania	123 685	371.3	479.6	210 456	70.2	145 403	451.2	559.6	248 959	71.2	269 088	409.0	519.6	459 415	70.7
HDI of countries and territories															
Low	474 370	122.7	40.6	1 160 337	144.6	337 841	98.9	28.0	806 151	138.6	812 211	110.6	34.3	1 966 488	142.1
Medium	1 261 351	114.2	70.8	2 445 324	93.9	1 162 894	111.6	58.5	2 383 754	105.0	2 424 245	112.3	64.5	4 829 078	99.2
High	3 612 996	181.0	181.9	5 546 725	53.5	3 823 126	198	170.9	6 652 363	74.0	7 436 122	187.5	176.3	12 199 088	64.1
Very high	4 312 457	261.9	419.7	5 681 725	31.8	4 983 714	320.6	478.2	7 487 212	50.2	9 296 171	285.7	448.6	13 168 937	41.7

Abbreviations: ASIR, age-standardized incidence rate; HDI, Human Development Index.

^a^ Estimates for ASIR are per 100 000 persons and were adjusted using the World Standard Population.

^b^ The number of cases diagnosed in 2022 and survived in the same year, as well as the 5-year period prevalence, is presented in eTable 2 in Supplement 1.

**Table 3.** Distribution of Cancer Deaths, Rates, and MIR in 2022 and Projected Deaths by 2050 Across Age, HDI, and Region

Characteristic	Females					Males					Both sexes				
	No. of deaths in 2022	ASMR^a^	MIR^b^	No. of deaths in 2050	Change, %	No. of deaths in 2022	ASMR^a^	MIR^b^	No. of deaths in 2050	Change, %	No. of deaths in 2022	ASMR^a^	MIR^b^	No. of deaths in 2050	Change, %
Age group, y															
0-19	44 650	3.6	37.1	46 502	4.2	60 695	4.6	39.7	61 689	1.6	105 345	4.1	38.3	108 191	2.7
20-39	195 992	16.1	24.0	221 406	13.0	155 118	12.4	36.3	175 010	12.8	351 110	14.2	28.2	396 416	12.9
40-64	1 489 485	134.9	35.4	2 026 682	36.1	1 773 090	163.3	48.2	2 527 742	42.6	3 262 575	148.8	41.4	4 554 423	39.6
65-74	1 072 140	409.1	48.1	1 792 872	67.2	1 616 008	690.8	50.5	2 783 908	72.3	2 688 148	542.0	49.5	4 576 780	70.3
≥75	1 511 281	837.4	64.7	5 694 788	120.4	1 825 373	1454.4	64.3	7 726 482	124.5	3 336 654	1090.9	64.3	13 421 270	122.8
Region															
Africa	416 898	89.8	63.8	1 010 342	142.4	346 945	89.9	71.5	873 980	151.9	763 843	88.9	67.2	1 884 322	146.7
Asia	2 270 297	70.5	44.7	4 448 116	95.9	3 194 154	107.7	61.8	6 320 955	97.9	5 464 451	88.0	53.5	10 769 071	97.1
Europe	894 222	84.4	33.3	1 150 917	28.7	1 091 871	135.3	42.3	1 558 850	42.8	1 986 093	106.3	38.0	2 709 767	36.4
Latin America and the Caribbean	365 838	77.6	43.7	709 467	93.9	383 404	96.5	48.3	794 801	107.3	749 242	85.5	46.0	1 504 267	100.8
Northern America	332 967	74.9	22.0	524 789	57.6	373 460	95.1	23.9	641 750	71.8	706 427	83.9	23.0	1 166 539	65.1
Oceania	33 326	82.9	22.3	64 063	92.2	40 450	106.0	23.5	80 026	97.8	73 776	93.4	22.8	144 089	95.3
HDI of countries and territories															
Low	306 630	82.8	67.5	764 968	149.5	237 970	72.2	73.0	575 001	141.6	544 600	77.3	69.9	1 339 969	146.1
Medium	764 722	69.9	61.2	1 588 698	107.8	795 332	77.0	69.0	1 678 918	111.1	1 560 054	73.1	65.1	3 267 616	109.5
High	1 605 339	72.4	40.0	3 067 344	91.1	2 385 933	119.9	60.6	4 570 114	91.5	3 991 272	94.5	50.4	7 637 458	91.4
Very high	1 634 910	78.6	30.0	2 420 685	48.1	2 008 592	118.3	36.9	3 292 383	63.9	3 643 502	96.0	33.6	5 713 068	56.8

Abbreviations: ASMR, age-standardized mortality rate; HDI, Human Development Index; MIR, mortality to incidence ratio.

^a^ Estimates for ASMR are per 100 000 persons and were adjusted using the World Standard Population.

^b^ The number of cases diagnosed in 2022 and survived in the same year, as well as the 5-year period prevalence, is presented in eTable 2 in Supplement 1.

In 2022, the global ASIR and ASMR per 100 000 persons were 196.9 cases and 91.7 deaths, respectively, with variations observed across regions. The highest and lowest ASIRs were observed in Oceania (409.0 cases per 100 000 persons) and Africa (132.3 cases per 100 000 persons), respectively. The ASMR ranged from 106.3 deaths per 100 000 in Europe to 83.9 deaths per 100 000 in North America. In 2050, cancer cases and deaths are projected to increase in all regions, yet Africa is expected to have a more than 5-fold increase compared with Europe (the region with the lowest increase): 139.4% vs 24.6% for cases (Table 2) and 146.7% vs 36.4% for deaths (Table 3).

Across 185 countries and territories globally, ASIRs were not uniformly distributed, ranging from 35.9 cases per 100 000 persons in Sierra Leone to 462.5 cases per 100 000 persons in Australia (eFigure 1A and eTable 4 in Supplement 1). The highest and lowest ASMRs were in Mongolia (181.5 deaths per 100 000 persons) and Saudi Arabia and Qatar (46.2 deaths per 100 000 persons), respectively (eFigure 1B and eTable 5 in Supplement 1). Between 2022 and 2050, cancer cases and deaths are projected to increase in 181 of 185 countries and territories (97.8%), with decreases expected in the remaining countries (eg, both cases and deaths are expected to decline in Moldova and Serbia) (eTables 4 and 5 in Supplement 1). The prevalence of cancer per 100 000 persons in 2022 ranged from 18.4 cases in Niger to 711.0 cases in Australia (eTable 6 in Supplement 1). In about half of countries and territories, increases greater than 2-fold in cancer cases (44.9%) and deaths (56.8%) are expected, with the highest increase projected in Kuwait at 338.2% for cases and 544.4% for deaths (eTables 4 and 5 in Supplement 1).

### MIR in 2022

The global MIR in 2022 was 46.6%, with the highest MIR observed for pancreatic cancer (89.4%) and the lowest for thyroid cancer (4.8%). Of the 36 cancer types studied, 8 (22.2%) had an MIR lower than 30.0%, whereas the rest fell into 2 categories: those with an MIR ranging from 30.0% to 50.0% (13 types [36.1%]) and those with an MIR exceeding 50.0% (14 types [38.9%]) (Table 1). The 5 cancer types with the highest MIR were pancreatic, liver, esophageal, mesothelioma, and brain and central nervous system cancers (eTable 3 in Supplement 1). The MIR was 10.4% higher in males than females (51.7% vs 41.3%) (Table 1).

The MIR was higher in the extreme age groups: 38.3% among individuals aged 19 years or younger and 64.3% among those 75 years or older, with the lowest MIR observed among the group aged 20 to 39 years (28.2%). Across age groups, males had a slightly higher MIR than females, with the largest gap observed among the groups aged 20 to 39 years (36.3% vs 24.0%) and 40 to 64 years (48.2% vs 35.4%) (Table 3).

There was an inverse association between MIR and HDI, with low-HDI countries experiencing an MIR (69.9%) nearly double that of very high–HDI countries (33.6%). Africa recorded the highest MIR at 67.2%, whereas Oceania had the lowest at 22.8% (Table 3).

**Figure 2.**
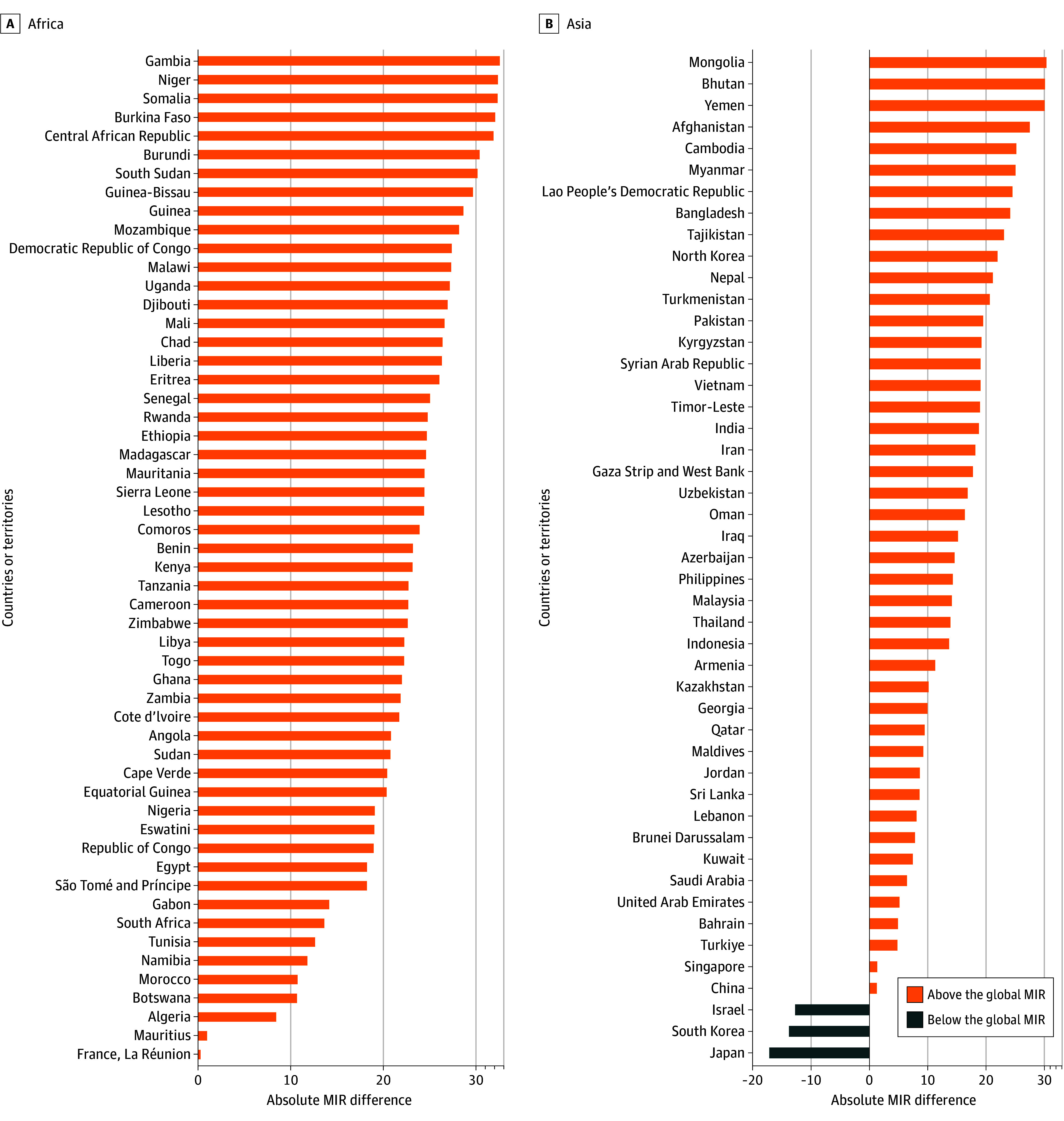
Differences in Mortality to Incidence Ratios (MIRs) in Each Country or Territory of 2 Regions Compared With the Global MIR, 2022 Data are presented for countries in Africa and Asia. Absolute MIR differences between each country or territory and the global values were calculated by subtracting the global MIR from each country or territory value. The negative values are indicated below (lower than) the global values (46.6%).

A 4-fold difference in MIR was also seen across countries and territories, ranging from 18.3% in Australia to 79.2% in the Republic of the Gambia. Of the 185 countries, 123 (66.5%) reported an MIR greater than 50.0%, 48 countries (25.9%) had an MIR ranging between 30.0% and 50.0%, and 14 countries (7.6%) had an MIR below 30.0% (eTable 7 in Supplement 1). The countries with the highest MIRs included the Republic of the Gambia, Niger, Somalia, Burkina Faso, and the Central African Republic. Rwanda has an MIR of 71.4%, which was lower than 25 low-HDI countries (eTable 7 in Supplement 1). Nearly three-fourths of countries and territories (136 [73.5%]) had an MIR higher than the global MIR (Figure 2 and eFigure 2 and eTable 7 in Supplement 1). A quarter of countries and territories (47 [25.4%]) had MIR values of 1.5 to 1.7 (ratios of MIR), as high as the global value (eTable 7 in Supplement 1). All 54 countries and territories in Africa had an MIR higher than the global estimate (Figure 2A), whereas 30 of the 40 European countries and territories (75.0%) exhibited an MIR lower than the global MIR (eFigure 2B in Supplement 1).

## Discussion

This study used population-based data from 185 countries and territories worldwide to describe the epidemiology of 36 cancers in 2022 and projections for 2050. We observed variations in cancer burden based on cancer type, sex, age, HDI, region, and specific countries and territories. A 3-fold increase in incident cancer cases (142.1% increase) and deaths (146.1% increase) is projected in low-HDI countries by 2050, compared with a 41.7% increase for cases and 56.8% for deaths in very high–HDI countries, highlighting the growing divide in the global cancer burden. Cancer incidence and mortality rates varied by region, with an increase expected in 181 of the 185 countries and territories in this study (97.8%) from 2022 to 2050. Approximately half of these countries are projected to see their cancer incidence and death rates double, with Kuwait experiencing the largest increase in both incidence and death rates. In this study, lung cancer was the leading cancer in terms of the number of incident cases and deaths, and it is expected to remain so by 2050, accounting for over 10% of all cases and deaths. Higher MIRs, indicating lower survival rates, were noted for less common and rare cancers such as mesothelioma, pancreatic, liver, and esophageal cancers. We found that low- and medium-HDI countries had MIRs nearly double those of very high–HDI countries, suggesting a gap in the availability of prevention, early detection, and optimal treatment services. The MIR was found to be 10.4% higher in males than in females, with projections by 2050 indicating a greater increase in cancer cases (15.8%) and deaths (8.0%) among males than females. Despite incidence and mortality rate increasing with age, the MIR was higher in the extreme age groups (≤19 or ≥75 years).

Cancer prevention and health promotion strategies play a vital role in mitigating the global cancer burden by addressing modifiable risk factors such as tobacco use, alcohol use, overweight, and exposure to carcinogens and UV radiation, alongside promoting healthy and balanced dietary choices, physical activity, vaccination, and screening uptake.^24,25,26,27^ Strengthening the development and implementation of tobacco and alcohol control measures (including taxation, advertising bans, and smoke-free policies) and promoting access to and consumption of healthy diets rich in fruits, vegetables, and whole grains while limiting processed foods and saturated fats have been shown to reduce cancer risk.^24,25,26,27^ Expansion of community-based screening programs will be important for prevention, early detection, and reduction of cancer-related morbidity and mortality.^24,25,26,27^ These programs can be expanded through various options, including educational campaigns using diverse platforms such as social media, print materials, and public workshops; scaling up trust and satisfaction by disseminating culturally acceptable and multilingual screening information; ensuring access by offering mobile screening options; and working closely with primary care providers for timely referral and service provision. Moreover, working closely with local organizations, government agencies, and advocacy groups to mobilize resources and shape policies that prioritize cancer prevention and promotion is essential.^24,25,26,27^

Expanding universal health insurance coverage and primary health care worldwide presents a promising strategy to reduce disparities and improve cancer outcomes through leveraging efforts for cancer prevention and providing basic cancer care options.^28,29^ However, there is a notable gap regarding universal health insurance coverage and access to primary health care between low- and high-HDI countries or within high-HDI countries, signaling the importance of sharing experiences within countries to achieve better cancer outcomes.^28,29^ For example, Rwanda, a low-HDI country, had a lower MIR compared with 25 low-HDI countries. This finding might be partially attributed to Rwanda’s more accessible universal health insurance coverage,^28^ and low-HDI countries could take the lessons from Rwanda to improve their efforts to expand universal health insurance coverage.^28^ Among high-HDI countries, Australia had the lowest MIR in this study, which could be attributed to its high ranking in health care system performance (measured by ensuring universal health insurance coverage and access and equity in primary health care).^29^ Hence, high-HDI countries could learn from Australia’s example in reducing cancer disparities.^29^

Compared with high-HDI countries, low- and medium-HDI countries experienced higher MIRs in this study, and disproportionately higher increases in cancer cases and deaths are projected. Many factors could contribute to this, such as increased life expectancy and aging, which are disproportionately affected by global emergencies and crises. Targeted cancer interventions could also be affected due to experiencing a double burden of disease and the associated competing priorities in resource allocation and cancer-targeted intervention.^27,30,31^ Low- and medium-HDI countries were among those with their cancer services highly disrupted by the COVID-19 pandemic and market crisis (including medical equipment) as a result of war, for example, in Ukraine, Yemen, Somalia, and Ethiopia.^1,30,32^ Between 2020 and 2021, the decline in numerical values of HDI due to a drop in HDI indicators within countries was approximately 2-fold greater in low- and medium-HDI countries (60%) compared with high-HDI countries (30%), signaling the importance of strengthening global efforts for pandemic preparedness and maintaining consistent cancer services.^1^ Promising and parallel reductions in common modifiable risk factors of cancer, such as smoking, also have not been observed in low-HDI countries compared with high-HDI countries, highlighting poor implementation of mass strategies.^31^ For instance, between 1990 and 2020, the worldwide smoking rate declined by 40%.^31^ However, this decline was uneven, with low- and medium-HDI countries experiencing a minimal decrease (<10%) or no reduction (eg, ≥50% continued smoking in Asia).^31^ This finding underscores the importance of strengthening cancer prevention efforts in low- and medium-HDI countries.^27,31^

Disparities by sex were observed in this study, with males having higher MIRs or cancer cases and deaths than females in 2022, and the variations in cases and deaths are projected to further widen by up to 15.8% in 2050. These disparities could be attributed to a complex interplay of factors. For example, compared with females, males are less likely to engage in cancer prevention activities, more likely to under use available screening and treatment options, and face a higher burden of modifiable risk factors such as smoking and alcohol consumption. Biological differences may also contribute.^31,33^ Although female-specific cancer screening programs, such as for breast and cervical cancer, have benefited females, there is a crucial lack of comparable programs for male-specific cancers such as prostate and testicular cancer.^33^ Furthermore, males participate less frequently in shared screening programs such as those for colorectal cancer.^33^ Males also have higher occupational exposure to carcinogens as well as a higher smoking rate than females (32.6% vs 6.5% in 2020).^31,34^

Higher incidence rates of cancer in high-HDI countries could be attributed to several interrelated factors, such as aging, sedentary behavior, consumption of highly processed foods, and high diagnostic rates.^35,36^ Although rates of smoking and alcohol consumption declined between 1990 and 2020, their previous exposure could lead to higher cancer cases.^31,35,36^ In this study, the observed higher MIR (indicative of better survival) among high-HDI countries, despite their high incidence rates, could be attributed to their advanced health care infrastructure.^24,25,26,27^

### Limitations

This study had some limitations, such as the quality of the GLOBOCAN data, including the potential cancer surveillance disruptions during COVID-19, that may influence the study’s estimates. This is particularly important for low- and middle-income countries with less robust cancer registries and civil registration systems.^14,15^ However, GLOBOCAN used various estimation strategies, including leveraging national data or modeling based on neighboring countries, to enhance the accuracy of estimates where possible.^12^ It is important to note that the current study’s findings align with prior population-based global research.^37,38^ Notably, the quality and coverage of cancer registry data sources have been improving over time, and continued efforts toward their expansion and maintenance are crucial for generating precise cancer outcome estimates worldwide.^14,39^

## Conclusions

In this cross-sectional study based on GLOBOCAN data from 2022, disparities were observed by HDI, region, cancer type, age, and sex, with inequities estimated to further widen by 2050. A higher MIR was observed for rare and less common cancer types, among males, by age group (≤19 or ≥75 years), and for low- and medium-HDI countries or territories. On the basis of these findings, cancer cases and deaths are projected to nearly triple in low-income countries by 2050 compared to a moderate increase in high-income countries (142.1% vs 41.7% for cancer cases and 146.1% vs 56.8% for cancer deaths). Greater increases in cancer cases (15.8%) and deaths (8.0%) are projected among males compared with females. Strengthening health care access and quality, including universal health insurance coverage, and health care systems in the prevention, early diagnosis, management, and treatment of cancer will be paramount for improving clinical outcomes and slowing projected trends.

## Notes

Supplement 1.
eMethods.
**eTable 1.** *International Classification of Diseases*, *Tenth Revision* (*ICD-10*), Codes for Cancers **eTable 2.** Cancer Prevalence in 2022 and Over the Past 5 Years From 2018 to 2022, by Cancer Type, Age, Region, and Human Development Index **eTable 3.** Top-10 Ranked Cancers by Country or Territory and Various Epidemiological Measures (Cases, Deaths, ASIR, ASMR, Prevalence, and MIR) for 2022 and 2050 **eFigure 1.** Rates of Cancer in Each Country or Territory, 2022 **eTable 4.** Distribution of Cancer Cases and Incidence Rate in 2022 and the Projected Cases and Percentage of Change by 2050 in Each Country or Territory Sorted by Alphabetical Order **eTable 5.** Distribution of Cancer Deaths and Mortality Rate in 2022 and the Projected Deaths and Percentage of Change by 2050 in Each Country or Territory Sorted by Alphabetical Order **eTable 6.** Cancer Prevalence in 2022 and Over the Past 5 Years (2018-2022) by Country or Territory **eTable 7.** Distribution of Cancer Mortality to Incidence Ratio (MIR), Absolute Differences, and Ratios Compared to the Global MIR in 2022 in Each Country or Territory Sorted by Alphabetical Order **eFigure 2.** Differences in Mortality to Incidence Ratios (MIRs) in Each Country or Territory of 3 Regions Compared With the Global MIR, 2022
eReferences

Supplement 2.
Data Sharing Statement
