# Supplementary material for: Global Disparities of Cancer and Its Projected Burden in 2050
Source: JAMA Netw Open. 2024 Nov 5;7(11):e2443198. doi: 10.1001/jamanetworkopen.2024.43198 (PMC11539015; doi:10.1001/jamanetworkopen.2024.43198)
Supplement: Supplement 1. — eMethods. eTable 1. International Classification of Diseases, Tenth Revision (ICD-10), Codes for Cancers eTable 2. Cancer Prevalence in 2022 and Over the Past 5 Years From 2018 to 2022, by Cancer Type, Age, Region, and Human Development Index eTable 3. Top-10 Ranked Cancers by Country or Territory and Various Epidemiological Measures (Cases, Deaths, ASIR, ASMR, Prevalence, and MIR) for 2022 and 2050 eFigure 1. Rates of Cancer in Each Country or Territory, 2022 eTable 4. Distribution of Cancer Cases and Incidence Rate in 2022 and the Projected Cases and Percentage of Change by 2050 in Each Country or Territory Sorted by Alphabetical Order eTable 5. Distribution of Cancer Deaths and Mortality Rate in 2022 and the Projected Deaths and Percentage of Change by 2050 in Each Country or Territory Sorted by Alphabetical Order eTable 6. Cancer Prevalence in 2022 and Over the Past 5 Years (2018-2022) by Country or Territory eTable 7. Distribution of Cancer Mortality to Incidence Ratio (MIR), Absolute Differences, and Ratios Compared to the Global MIR in 2022 in Each Country or Territory Sorted by Alphabetical Order eFigure 2. Differences in Mortality to Incidence Ratios (MIRs) in Each Country or Territory of 3 Regions Compared With the Global MIR, 2022 eReferences [file jamanetwopen-e2443198-s001.pdf]

Supplementary Online Content

Bizuayehu HM, Ahmed KY, Kibret GD, et al. Global disparities of cancer and its projected burden in 2050. *JAMA Netw Open*. 2024;7(11):e2443198. doi:10.1001/jamanetworkopen.2024.43198

eMethods

eTable 1. *International Classification of Diseases*, *Tenth Revision* (*ICD-10*), Codes for Cancers

eTable 2. Cancer Prevalence in 2022 and Over the Past 5 Years From 2018 to 2022, by Cancer Type, Age, Region, and Human Development Index

eTable 3. Top-10 Ranked Cancers by Country or Territory and Various Epidemiological Measures (Cases, Deaths, ASIR, ASMR, Prevalence, and MIR) for 2022 and 2050

eFigure 1. Rates of Cancer in Each Country or Territory, 2022

eTable 4. Distribution of Cancer Cases and Incidence Rate in 2022 and the Projected Cases and Percentage of Change by 2050 in Each Country or Territory Sorted by Alphabetical Order

eTable 5. Distribution of Cancer Deaths and Mortality Rate in 2022 and the Projected Deaths and Percentage of Change by 2050 in Each Country or Territory Sorted by Alphabetical Order

eTable 6. Cancer Prevalence in 2022 and Over the Past 5 Years (2018-2022) by Country or Territory

eTable 7. Distribution of Cancer Mortality to Incidence Ratio (MIR), Absolute Differences, and Ratios Compared to the Global MIR in 2022 in Each Country or Territory Sorted by Alphabetical Order

eFigure 2. Differences in Mortality to Incidence Ratios (MIRs) in Each Country or Territory of 3 Regions Compared With the Global MIR, 2022

eReferences

This supplementary material has been provided by the authors to give readers additional information about their work.

© 2024 Bizuayehu HM et al. *JAMA Network Open.*

eMethods

Data sources

This study utilised population-based cancer data from GLOBOCAN 2022, curated by the International Agency for Research on Cancer. Detailed explanations of the GLOBOCAN dataset can be found in previously published studies.[1-3] The GLOBOCAN repository aggregates global cancer-related data, encompassing data from each country/territory. At the national level, GLOBOCAN estimates cancer cases, deaths, rates and prevalence using population-based administrative data sources such as cancer registries, civil and vital statistics registration systems, or modelling, applying robust methodologies tailored to the specific context of each country/territory.[1,2,4,5] Ethical approval and patient consent was not required for this study because the data used were publicly available.

Measures

This study included all cancer types available in the GLOBOCAN database, totalling 36 cancer groups: 1) 34 cancers individually, 2) all cancers combined, and 3) all cancers excluding nonmelanoma skin cancer. Specific cancer types were identified by referencing the International Classification of Diseases, 10th Revision (ICD-10) codes.[2,6] The ICD-10 codes relevant to our research are provided in the supplementary material (eTable 1). The dataset is further stratified by sex, age, country/territory, region, and HDI.[2,6,7] Age was grouped as 0-19, 20-39, 40-64, 65-74 and 75+ years based on its importance for epidemiology, policy, public health and clinical practice.[8,9] Data is available for seven regions and 185 countries/territories worldwide. In line with the 2022 United Nations Development Programme classification, HDI was reported in four tiers, i.e. low, medium, high, and very high.[7] Countries/territories were assigned to one of six

© 2024 Bizuayehu HM et al. *JAMA Network Open.*

regions – Africa, Asia, Europe, Latin America and the Caribbean, Northern America, and Oceania.

Statistical analysis

We reported various measures of burden, including counts, rates, prevalence, and MIR. Incidence and mortality rates were determined by dividing the number of cases and deaths respectively in 2022 by the total population in the same year, with age-standardised incidence rates (ASIR) and age-standardised mortality rates (ASMR) calculated by adjusting their crude rates with the world standard population, computed by Segi-Doll in 1966, with the Segi-Doll 1966 world standard population method used to facilitate comparison with previous literature.[2,10,11] To estimate MIR, the ASMR was divided by the ASIR and multiplied by 100 to obtain a percentage, with a higher MIR indicating poorer survival after a cancer diagnosis.[12-14] The absolute MIR difference between each country/territory and the global values was calculated by subtracting the global MIR from MIRs of each country/territory value (Absolute Difference in MIR= $MIR_{country/territory} - MIR_{global}$). The ratios of MIR between each country/territory and the global value were calculated by dividing the MIR value of each country/territory by the global value (Ratio of MIR= $MIR_{country/territory} / MIR_{global}$). Cancer prevalence was calculated by dividing the number of persons diagnosed with cancer and known to be alive in a specific period by the total population during that period. Specifically, this study calculated the point prevalence for 2022 (those diagnosed with cancer in 2022 and alive in the same year) and the five-year period prevalence (those diagnosed with cancer between 2018 and 2022 and alive in 2022).[1,11] To project future cancer cases and deaths, demographic projections were employed, assuming that the 2022 cancer rates remain stable.[3,15,16] Hence, the 2050 cancer estimates are generated by applying the 2022 standardised rates to the 2050 population predicted by the United Nations Development Programme.[7] The percentage

© 2024 Bizuayehu HM et al. *JAMA Network Open.*

changes in cancer cases were calculated by dividing the projected estimates between 2022 and 2050 by observed cases in 2022. A similar approach was followed to calculate changes in cancer deaths.[3,15,16] We calculated MIR, absolute MIR difference and ratios of MIR, while other estimates such as rates, prevalence, counts and projections were extracted from the GLOBOCAN *(https://gco.iarc.who.int)*; this approach is in line with existing studies on the same data source.[3,14-21] Each estimate was available by age structure in five-year intervals, i.e. 0-4, 5-9, …, 80-84, 85+. To achieve the study objective, specific estimates have been calculated, extracted, merged and/or appended. Data analysis was carried out using R,[22] Microsoft Excel and the Global Cancer Observatory's online tabulation and visualisation tools.

eTable 1. *International Classification of Diseases*, *Tenth Revision* (*ICD-10*), Codes for Cancers

ICD-10 Code	Cancer name
C00-97	All cancers
C00-97/C44	All cancers, excluding non-melanoma skin cancer
C67	Bladder
C50	Breast
C70-72	Brain, central nervous system
C53	Cervix uteri
C18-C21	Colorectum
C54	Corpus uteri
C23	Gallbladder
C81	Hodgkin lymphoma
C12-13	Hypopharynx
C46	Kaposi sarcoma
C64	Kidney
C32	Larynx
C91-95	Leukaemia
C00-06	Lip, oral cavity
C22	Liver and intrahepatic bile ducts
C43	Melanoma of skin
C45	Mesothelioma
C90	Multiple myeloma
C11	Nasopharynx
C82-86, C88	Non-Hodgkin lymphoma
C44	Non-melanoma skin cancer
C15	Oesophagus
C09-10	Oropharynx
C56	Ovary
C25	Pancreas
C60	Penis
C61	Prostate
C07-08	Salivary glands
C16	Stomach
C62	Testis
C73	Thyroid
C33-34	Trachea, bronchus and lung
C52	Vagina
C51	Vulva

© 2024 Bizuayehu HM et al. *JAMA Network Open.*

eTable 2. Cancer Prevalence in 2022 and Over the Past 5 Years From 2018 to 2022, by Cancer Type, Age, Region, and Human Development Index

	Females			Males			Both sexes		
	**2022[a]**	**2018-2022**		**2022[a]**	**2018-2022**		**2022[a]**	**2018-2022**	
	**Survivors[b]**	**Survivors[c]**	**Prevalence[d]**	**Survivors[b]**	**Survivors[c]**	**Prevalence[d]**	**Survivors[b]**	**Survivors[c]**	**Prevalence[d]**
**Cancer type**									
All cancers	7,004,087	27,756,915	709.5	7,103,954	25,747,272	648.1	14,108,041	53,504,187	678.6
All cancers, excluding NMSC	6,573,371	26,056,966	666.0	6,460,610	23,265,046	585.6	13,033,981	49,322,012	625.5
Lip, oral cavity	85,868	349,727	8.9	187,130	744,721	18.8	272,998	1,094,448	13.9
Salivary glands	18,330	78,433	2.0	23,458	92,137	2.3	41,788	170,570	2.2
Oropharynx	15,168	57,356	1.5	65,001	248,886	6.3	80,169	306,242	3.9
Nasopharynx	24,459	103,630	2.7	62,531	255,930	6.4	86,990	359,560	4.6
Hypopharynx	8,454	27,801	0.71	44,204	132,185	3.3	52,658	159,986	2.0
Oesophagus	86,264	209,930	5.4	220,572	507,239	12.8	306,836	717,169	9.1
Stomach	187,414	584,178	14.9	355,204	1,042,265	26.2	542,618	1,626,443	20.6
Colorectum	656,381	2,584,025	66.1	821,458	3,183,756	80.1	1,477,839	5,767,781	73.2
Liver	140,025	344,009	8.8	323,658	819,714	20.6	463,683	1,163,723	14.8
Gallbladder	44,854	106,934	2.7	25,496	61,019	1.5	70,350	167,953	2.1
Pancreas	108,743	214,338	5.5	123,592	247,141	6.2	232,335	461,479	5.9
Larynx	16,671	69,733	1.8	122,093	514,135	12.9	138,764	583,868	7.4
Lung	494,661	1,323,226	33.8	783,326	1,898,235	47.8	1,277,987	3,221,461	40.9
Melanoma of skin	138,740	597,976	15.3	163,830	664,780	16.7	302,570	1,262,756	16.0
NMSC	430,716	1,699,949	43.5	643,344	2,482,226	62.5	1,074,060	4,182,175	53.0
Mesothelioma	5,439	12,157	0.31	11,943	24,506	0.62	17,382	36,663	0.46
Kaposi sarcoma	6,346	24,946	0.64	14,616	57,176	1.4	20,962	82,122	1.0
Breast	1,871,979	8,178,393	209.0	NA	NA	NA	1,871,979	8,178,393	209.0
Vulva	36,962	142,049	3.6	NA	NA	NA	36,962	142,049	3.6
Vagina	13,078	46,755	1.2	NA	NA	NA	13,078	46,755	1.2
Cervix uteri	463,865	1,948,521	49.8	NA	NA	NA	463,865	1,948,521	49.8
Corpus uteri	351,256	1,594,042	40.7	NA	NA	NA	351,256	1,594,042	40.7
Ovary	237,176	929,996	23.8	NA	NA	NA	237,176	929,996	23.8
Penis	NA	NA	NA	27,607	110,717	2.8	27,607	110,717	2.8
Prostate	NA	NA	NA	1,212,426	5,033,178	126.7	1,212,426	5,033,178	126.7
Testis	NA	NA	NA	61,969	297,454	7.5	61,969	297,454	7.5
Kidney	120,427	506,110	12.9	216,299	863,864	21.7	336,726	1,369,974	17.4
Bladder	110,063	431,627	11.0	381,180	1,518,688	38.2	491,243	1,950,315	24.7
Brain, central nervous system	114,159	553,087	14.1	134,492	646,226	16.3	248,651	1,199,313	15.2
Thyroid	491,368	2,213,219	56.6	161,567	698,163	17.6	652,935	2,911,382	36.9
Hodgkin lymphoma	26,383	120,267	3.1	38,022	171,989	4.3	64,405	292,256	3.7
Non-Hodgkin lymphoma	182,732	769,035	19.7	233,923	969,481	24.4	416,655	1,738,516	22.1
Multiple myeloma	65,068	245,739	6.3	79,125	293,209	7.4	144,193	538,948	6.8
Leukaemia	149,650	617,213	15.8	203,971	837,372	21.1	353,621	1,454,585	18.5
**Age groups**									
**0-19**	86,820	399,154	31.7	110,833	525,610	39.2	197,653	924,764	35.6

© 2024 Bizuayehu HM et al. *JAMA Network Open.*

	Females			Males			Both sexes		
	2022[a]	2018-2022		2022[a]	2018-2022		2022[a]	2018-2022	
	Survivors[b]	Survivors[c]	Prevalence[d]	Survivors[b]	Survivors[c]	Prevalence[d]	Survivors[b]	Survivors[c]	Prevalence[d]
**20-39**	624,682	2,775,910	244.7	317,178	1,370,103	114.8	941,860	4,146,013	178.1
**40-64**	2,596,003	10,065,492	931.5	3,188,710	13,574,337	1251.0	5,784,713	23,639,829	1091.5
**65-74**	1,619,857	6,126,375	2360.7	2,237,227	7,992,976	3438.5	3,857,084	14,119,351	2870.0
**75+**	1,484,018	4,881,139	2779.6	1,842,713	5,793,091	4584.6	3,326,731	10,674,230	3534.9
**Regions**									
**Africa**	391,291	1,576,013	224.0	283,225	1,035,465	147.3	674,516	2,611,478	185.6
**Asia**	3,220,396	12,552,780	552.1	3,179,527	10,877,129	458.1	6,399,923	23,429,909	504.1
**Europe**	1,688,550	6,757,505	1749.3	1,821,587	6,888,582	1906.9	3,510,137	13,646,087	1825.5
**Latin America and the Caribbean**	555,676	2,207,518	652.6	518,451	1,888,514	577.2	1,074,127	4,096,032	615.5
**Northern America**	1,043,378	4,230,981	2244.1	1,178,592	4,568,584	2472.4	2,221,970	8,799,565	2357.1
**Oceania**	104,796	432,118	1977.4	122,572	488,998	2232.7	227,368	921,116	2105.2
**HDI of Countries and Territories**									
**Low**	244,368	984,182	163.6	170,390	630,328	103.4	414,758	1,614,510	133.3
**Medium**	785,130	3,102,053	279.6	678,632	2,425,622	209.1	1,463,762	5,527,675	243.6
**High**	2,490,575	9,678,940	706.9	2,375,479	7,987,283	574.5	4,866,054	17,666,223	640.2
**Very HDI**	3,484,014	13,991,740	1685.4	3,879,453	14,704,039	1812.3	7,363,467	28,695,779	1748.1

[a] The prevalence in 2022 has been reported in Tables 1 and 2.
[b] The number of cancer survivors who diagnosed with cancer in 2022
[c] The number of cancer survivors who diagnosed with cancer over the five-year span (2018-2022)
[d] The five-year prevalence per 100,000 persons
NA Not applicable

© 2024 Bizuayehu HM et al. *JAMA Network Open.*

eTable 3. Top-10 Ranked Cancers by Country or Territory and Various Epidemiological Measures (Cases, Deaths, ASIR, ASMR, Prevalence, and MIR) for 2022 and 2050

Top ten cancer types in 2022 by prevalence and MIR and their projected number of cases and deaths in 2050

	Females				Males				Both sexes			
**Rank**	**Prevalence**	**MIR**	**Cases**	**Deaths**	**Prevalence**	**MIR**	**Cases**	**Deaths**	**Prevalence**	**MIR**	**Cases**	**Deaths**
**1**	Breast	Pancreas	Breast	Lung	Prostate	Pancreas	Lung	Lung	Breast	Pancreas	Lung	Lung
**2**	Colorectum	Liver	Lung	Breast	Colorectum	Liver	Prostate	Colorectum	Prostate	Liver	Colorectum	Colorectum
**3**	Lung	Oesophagus	Colorectum	Colorectum	Lung	Oesophagus	Colorectum	Prostate	Colorectum	Oesophagus	Breast	Liver
**4**	Thyroid	Mesothelioma	NMSC	Cervix uteri	NMSC	Mesothelioma	NMSC	Liver	Lung	Mesothelioma	Prostate	Stomach
**5**	Cervix uteri	Gallbladder	Cervix uteri	Stomach	Bladder	BNS	Stomach	Stomach	NMSC	BNS	NMSC	Breast
**6**	NMSC	BNS	Thyroid	Pancreas	Stomach	Lung	Liver	Oesophagus	Cervix uteri	Lung	Stomach	Prostate
**7**	Corpus uteri	Stomach	Corpus uteri	Liver	Liver	Gallbladder	Bladder	Pancreas	Corpus uteri	Gallbladder	Liver	Pancreas
**8**	Ovary	Multiple myeloma	Stomach	Ovary	NHL	Stomach	Oesophagus	Bladder	Thyroid	Stomach	Bladder	Oesophagus
**9**	Stomach	Lung	Ovary	Oesophagus	Oesophagus	Nasopharynx	NHL	Leukaemia	Stomach	Multiple myeloma	Thyroid	Cervix uteri
**10**	NHL	Ovary	Liver	Leukaemia	Kidney	Multiple myeloma	Pancreas	NHL	Bladder	Ovary	Pancreas	Leukaemia

Top ten countries/territories in 2022 by ASIR, ASMR and MIR

	Females			Males			Both sexes		
**Rank**	**ASIR**	**ASMR**	**MIR**	**ASIR**	**ASMR**	**MIR**	**ASIR**	**ASMR**	**MIR**
**1**	Australia	Zimbabwe	CAR	Australia	Mongolia	Gambia	Australia	Mongolia	Niger
**2**	New Zealand	Mongolia	Somalia	New Zealand	Belarus	Niger	New Zealand	Zimbabwe	Somalia
**3**	Denmark	Malawi	Burkina Faso	USA	Hungary	Yemen	Denmark	Hungary	Burkina Faso
**4**	USA	Papua New Guinea	Burundi	Hungary	Lithuania	Somalia	USA	Poland	CAR
**5**	Norway	Fiji	Chad	Denmark	Romania	Mongolia	Norway	Romania	Burundi
**6**	The Netherlands	Uganda	South Sudan	Lithuania	Latvia	Burkina Faso	Canada	Serbia	Mongolia
**7**	Canada	Mali	Guinea-Bissau	France (metropolitan)	Moldova	Bhutan	Ireland	Uruguay	South Sudan
**8**	Ireland	Samoa	Niger	Ireland	Poland	Cambodia	The Netherlands	Samoa	Bhutan
**9**	Belgium	Zambia	Mozambique	Norway	Croatia	Lao PDR	France (metropolitan)	Croatia	Yemen
**10**	France (metropolitan)	Kenya	Malawi	Croatia	Türkiye	CAR	Hungary	Slovakia	Guinea-Bissau

Top ten countries or Territories with highest projected increases in cancer cases and deaths by 2050

	Females		Males		Both sexes	
**Rank**	**Cases change (%)[a]**	**Deaths change (%)[b]**	**Cases change (%)[a]**	**Deaths change (%)[b]**	**Cases change (%)[a]**	**Deaths change (%)[b]**
**1**	Kuwait	Kuwait	Kuwait	Kuwait	Kuwait	Kuwait
**2**	Equatorial Guinea	United Arab Emirates	United Arab Emirates	Bahrain	United Arab Emirates	United Arab Emirates
**3**	United Arab Emirates	Equatorial Guinea	Maldives	United Arab Emirates	Equatorial Guinea	Bahrain
**4**	Syrian Arab Republic	Qatar	Bahrain	Maldives	Syrian Arab Republic	Maldives
**5**	Tanzania	Bahrain	Cape Verde	Saudi Arabia	Bahrain	Saudi Arabia
**6**	Zambia	Syrian Arab Republic	Equatorial Guinea	Cape Verde	Zambia	Equatorial Guinea
**7**	Niger	Saudi Arabia	Saudi Arabia	Equatorial Guinea	Tanzania	Syrian Arab Republic
**8**	Burundi	Mongolia	Zambia	French Guyana	Maldives	Zambia
**9**	Angola	Iran	Tanzania	Zambia	Niger	Cape Verde
**10**	Gaza Strip and West Bank	Gaza Strip and West Bank	Syrian Arab Republic	Syrian Arab Republic	Saudi Arabia	Tanzania

[a] Cases change in percentage refers the proportional change in cases between 2022 and 2050, with countries/territories in rank one will have the highest projected proportional increase in cancer cases by 2050

[b] Deaths change in percentage refers the proportional change in deaths between 2022 and 2050, with countries/territories in rank one will have the highest projected proportional increase in cancer deaths by 2050

© 2024 Bizuayehu HM et al. *JAMA Network Open.*

ASIR=Age-Standardised Incidence Rate, ASMR=Age-Standardised Mortality Rate, BNS=Brain Central Nervous System, CAR=Central African Republic, Lao PDR=Lao People's Democratic Republic, MIR=Mortality-to-Incidence Ratio, NHL=Non-Hodgkin Lymphoma, NMSC=Non-Melanoma Skin Cancer, USA=United States of America.

© 2024 Bizuayehu HM et al. *JAMA Network Open.*

eFigure 1. Rates of Cancer in Each Country or Territory, 2022

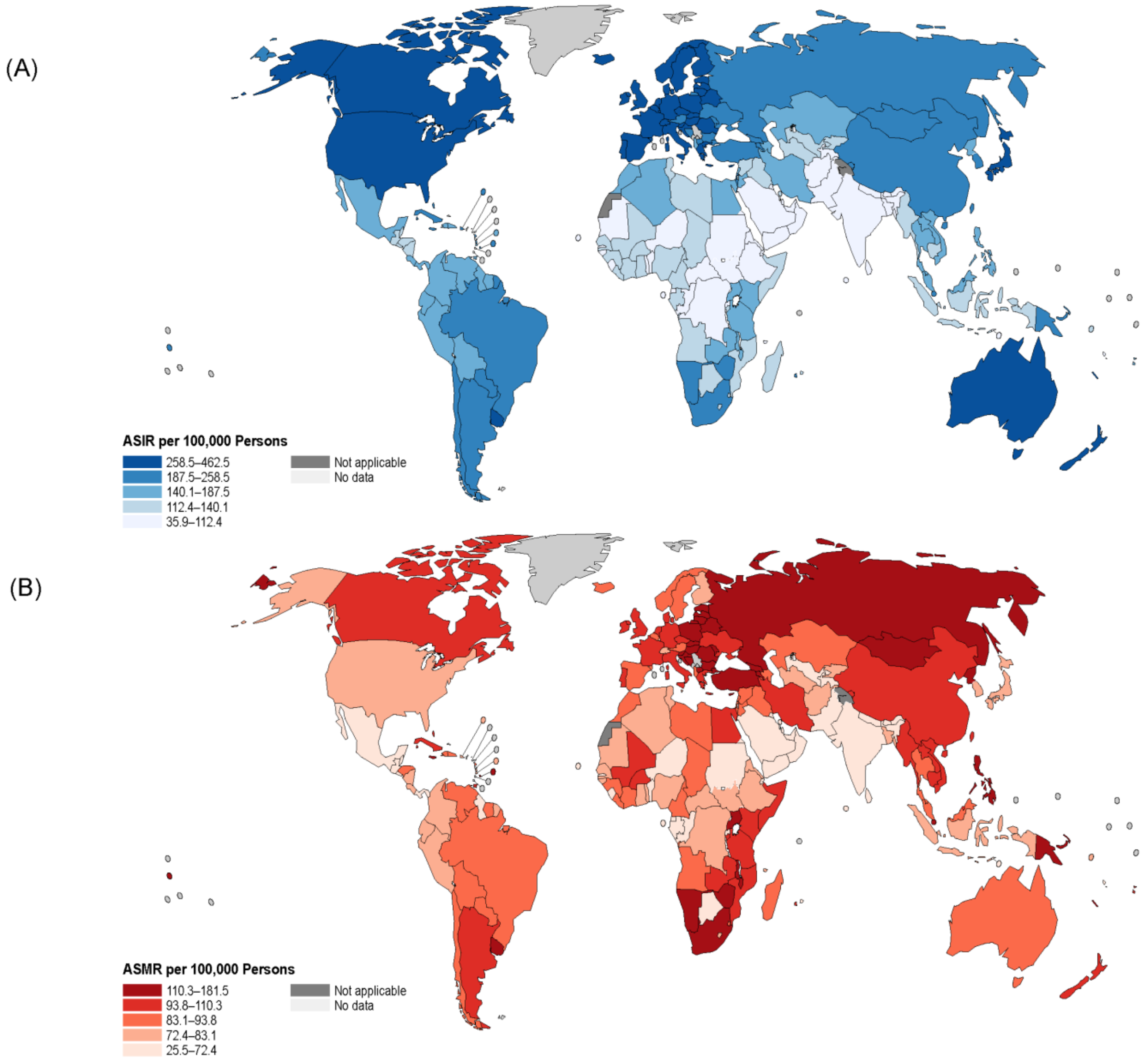

A, Age-standardized incidence rate (ASIR). B, Age-standardized mortality rate (ASMR).

© 2024 Bizuayehu HM et al. *JAMA Network Open.*

eTable 4. Distribution of Cancer Cases and Incidence Rate in 2022 and the Projected Cases and Percentage of Change by 2050 in Each Country or Territory Sorted by Alphabetical Order

Country or territory	Females				Males				Both sexes			
	2022 cases	ASIR[a]	2050 cases	Change (%)[b]	2022 cases	ASIR[a]	2050 cases	Change (%)[b]	2022 cases	ASIR[a]	2050 cases	Change (%)[b]
**Afghanistan**	13,036	110.7	34,662	165.9	11,239	103.6	27,144	141.5	24,275	106.2	61,806	154.6
**Albania**	3,432	147.7	4,533	32.1	4,587	176.9	6,475	41.2	8,019	160.8	11,008	37.3
**Algeria**	35,326	152.2	65,612	85.7	29,387	130.6	68,422	132.8	64,713	141.2	134,034	107.1
**Angola**	13,923	137.3	38,616	177.4	10,684	132.3	30,147	182.2	24,607	133.5	68,764	179.5
**Argentina**	68,380	208.7	102,482	49.9	65,040	231.8	117,193	80.2	133,420	215.8	219,675	64.7
**Armenia**	4,474	163.9	6,164	37.8	5,046	257.5	5,988	18.7	9,520	201.6	12,152	27.7
**Australia**	95,969	415.2	151,545	57.9	116,363	514.3	187,044	60.7	212,332	462.5	338,589	59.5
**Austria**	23,361	235.7	29,363	25.7	27,321	287.6	38,555	41.1	50,682	258.5	67,918	34.0
**Azerbaijan**	8,602	131.8	13,842	60.9	9,901	181.2	17,828	80.1	18,503	152.3	31,669	71.2
**Bahamas**	478	177.6	975	104.0	477	214.4	984	106.3	955	192.7	1,958	105.0
**Bahrain**	729	137.4	1,747	139.6	654	105.5	2,455	275.4	1,383	113.8	4,202	203.8
**Bangladesh**	72,334	89.5	156,574	116.5	94,922	120.8	191,209	101.4	167,256	105.6	347,783	107.9
**Barbados**	542	190.7	639	17.9	578	224.5	677	17.1	1,120	205.3	1,316	17.5
**Belarus**	21,469	223.6	23,491	9.4	24,933	360.5	31,808	27.6	46,402	273.2	55,299	19.2
**Belgium**	37,487	306.6	46,509	24.1	43,645	350.8	61,545	41.0	81,132	324.7	108,053	33.2
**Belize**	221	118.2	529	139.4	188	110.1	458	143.6	409	114.7	987	141.3
**Benin**	4,028	101.3	9,882	145.3	3,468	108.2	9,247	166.6	7,496	102.6	19,129	155.2
**Bhutan**	282	84.5	677	140.1	356	90.5	816	129.2	638	87.6	1,494	134.2
**Bolivia**	9,887	159.4	17,196	73.9	7,692	129.6	12,384	61.0	17,579	143.8	29,580	68.3
**Bosnia Herzegovina**	6,424	194.2	6,465	0.6	7,841	251.7	9,115	16.3	14,265	218.6	15,580	9.2
**Botswana**	1,376	119.4	2,730	98.4	941	114.1	2,324	147.0	2,317	115.4	5,054	118.1
**Brazil**	307,482	197.6	524,722	70.7	319,711	240.1	626,225	95.9	627,193	214.4	1,150,947	83.5
**Brunei Darussalam**	529	216.0	1,082	104.5	396	171.7	995	151.3	925	192.2	2,077	124.5
**Bulgaria**	15,297	203.2	13,482	-11.9	17,515	253.9	18,420	5.2	32,812	222.0	31,902	-2.8
**Burkina Faso**	8,982	138.5	23,102	157.2	5,556	112.5	15,614	181.0	14,538	125.2	38,716	166.3
**Burundi**	4,627	134.6	13,168	184.6	3,370	123.5	10,042	198.0	7,997	128.2	23,210	190.2
**Cambodia**	10,624	130.0	21,097	98.6	9,171	152.9	22,633	146.8	19,795	138.3	43,730	120.9
**Cameroon**	11,578	139.9	29,402	154.0	7,986	113.3	20,986	162.8	19,564	126.0	50,389	157.6
**Canada**	138,438	331.8	212,326	53.4	153,660	365.9	257,099	67.3	292,098	345.9	469,425	60.7
**Cape Verde**	221	78.3	476	115.4	214	108.6	705	229.4	435	87.0	1,182	171.7
**Central African Republic**	1,615	110.0	3,836	137.5	1,075	96.7	2,575	139.5	2,690	101.8	6,410	138.3
**Chad**	4,580	94.4	12,158	165.5	5,605	147.4	14,646	161.3	10,185	118.1	26,803	163.2
**Chile**	27,076	165.1	46,522	71.8	32,800	221.7	69,969	113.3	59,876	188.7	116,491	94.6
**China**	2,290,797	197.0	3,218,639	40.5	2,533,906	209.6	4,087,068	61.3	4,824,703	201.6	7,305,707	51.4
**Colombia**	61,396	175.3	111,192	81.1	56,224	183.3	117,160	108.4	117,620	177.6	228,351	94.1
**Comoros**	382	122.0	804	110.5	237	92.9	582	145.6	619	106.8	1,386	123.9
**Democratic Republic of Congo**	29,579	107.8	77,371	161.6	23,033	100.1	58,457	153.8	52,612	103.0	135,828	158.2
**Republic of Congo**	1,556	82.3	3,602	131.5	1,171	84.2	3,186	172.1	2,727	80.7	6,788	148.9
**Costa Rica**	6,517	173.5	11,901	82.6	6,808	185.5	14,503	113.0	13,325	177.7	26,405	98.2
**Côte d'Ivoire**	11,557	143.7	29,535	155.6	9,795	130.0	23,288	137.8	21,352	137.0	52,823	147.4
**Croatia**	13,202	277.0	12,714	-3.7	15,607	367.3	18,437	18.1	28,809	313.4	31,150	8.1
**Cuba**	22,796	199.1	29,520	29.5	26,892	247.4	36,009	33.9	49,688	220.8	65,529	31.9

© 2024 Bizuayehu HM et al. *JAMA Network Open.*

Country or territory	Females				Males				Both sexes			
	**2022 cases**	**ASIR[a]**	**2050 cases**	**Change (%)[b]**	**2022 cases**	**ASIR[a]**	**2050 cases**	**Change (%)[b]**	**2022 cases**	**ASIR[a]**	**2050 cases**	**Change (%)[b]**
**Cyprus**	3,128	300.1	5,095	62.9	3,070	289.9	6,144	100.1	6,198	292.3	11,240	81.4
**Czechia**	30,643	254.9	32,630	6.5	35,033	317.5	44,281	26.4	65,676	280.9	76,912	17.1
**Denmark**	23,014	361.2	28,814	25.2	25,826	394.0	34,765	34.6	48,840	374.7	63,579	30.2
**Djibouti**	494	111.3	1,089	120.5	311	71.6	634	103.9	805	90.7	1,724	114.2
**Dominican Republic**	9,436	150.5	17,020	80.4	10,735	186.7	20,421	90.2	20,171	167.0	37,441	85.6
**Ecuador**	16,998	163.5	35,275	107.5	13,890	143.3	32,329	132.8	30,888	152.7	67,604	118.9
**Egypt**	76,967	161.1	160,439	108.5	73,611	175.1	162,875	121.3	150,578	166.1	323,313	114.7
**El Salvador**	5,594	128.4	9,013	61.1	4,205	126.5	6,584	56.6	9,799	127.1	15,597	59.2
**Equatorial Guinea**	516	128.0	1,664	222.5	410	93.7	1,347	228.5	926	107.7	3,012	225.3
**Eritrea**	1,553	119.7	3,517	126.5	910	78.7	1,988	118.5	2,463	99.8	5,505	123.5
**Estonia**	3,836	237.9	3,848	0.3	4,214	356.4	5,632	33.7	8,050	282.5	9,480	17.8
**Eswatini**	764	173.6	1,624	112.6	344	107.8	781	127.0	1,108	135.3	2,406	117.2
**Ethiopia**	52,621	131.8	136,590	159.6	27,713	75.8	69,539	150.9	80,334	104.5	206,128	156.6
**Fiji**	947	201.1	1,444	52.5	654	150.3	985	50.6	1,601	174.8	2,429	51.7
**Finland**	17,923	266.7	20,489	14.3	19,737	294.9	25,116	27.3	37,660	276.9	45,605	21.1
**France (metropolitan)**	220,229	301.0	271,870	23.5	263,339	386.4	332,322	26.2	483,568	339.0	604,193	24.9
**France, Guadeloupe**	811	184.2	965	19.0	1,312	340.2	1,201	-8.5	2,123	253.9	2,166	2.0
**France, La Réunion**	1,245	163.9	2,107	69.2	1,770	248.7	2,900	63.8	3,015	203.1	5,007	66.1
**France, Martinique**	882	189.5	965	9.4	1,175	266.9	1,152	-2.0	2,057	224.2	2,117	2.9
**French Guyana**	248	163.6	577	132.7	341	250.5	841	146.6	589	204.3	1,418	140.8
**French Polynesia**	400	211.2	834	108.5	477	247.1	1,154	141.9	877	228.1	1,988	126.7
**Gabon**	1,133	134.6	2,633	132.4	742	96.4	1,820	145.3	1,875	114.4	4,453	137.5
**Gaza Strip and West Bank**	2,524	142.6	6,945	175.2	2,506	165.5	7,229	188.5	5,030	152.3	14,174	181.8
**Georgia**	6,436	165.5	6,481	0.7	7,253	246.8	7,346	1.3	13,689	197.5	13,827	1.0
**Germany**	282,260	249.0	331,383	17.4	323,545	306.5	419,521	29.7	605,805	274.2	750,905	24.0
**Ghana**	15,987	135.2	36,984	131.3	11,398	106.2	26,630	133.6	27,385	120.4	63,614	132.3
**Greece**	29,139	231.3	32,864	12.8	36,564	295.0	44,288	21.1	65,703	258.7	77,152	17.4
**Guam**	191	155.6	306	60.2	221	185.0	390	76.5	412	167.2	696	68.9
**Guatemala**	10,018	124.7	22,290	122.5	7,783	119.6	18,770	141.2	17,801	121.8	41,061	130.7
**Guinea**	5,622	124.0	13,179	134.4	3,155	108.9	9,651	205.9	8,777	116.3	22,830	160.1
**Guinea-Bissau**	742	112.7	1,825	146.0	428	87.4	1,197	179.7	1,170	100.3	3,022	158.3
**Guyana**	688	157.7	1,050	52.6	537	130.1	801	49.2	1,225	142.5	1,851	51.1
**Haiti**	6,832	127.8	12,155	77.9	7,028	161.3	12,143	72.8	13,860	141.1	24,298	75.3
**Honduras**	5,764	127.7	12,393	115.0	5,051	131.2	12,258	142.7	10,815	128.0	24,651	127.9
**Hungary**	32,090	298.8	32,965	2.7	34,250	395.9	42,605	24.4	66,340	336.7	75,570	13.9
**Iceland**	868	266.8	1,372	58.1	909	271.7	1,622	78.4	1,777	268.3	2,994	68.5
**India**	722,138	100.8	1,336,513	85.1	691,178	97.1	1,354,170	95.9	1,413,316	98.5	2,690,683	90.4
**Indonesia**	220,266	141.6	345,878	57.0	188,395	135.5	336,817	78.8	408,661	136.9	682,695	67.1
**Iran**	60,757	129.1	132,906	118.8	76,441	168.4	189,967	148.5	137,198	149.0	322,873	135.3
**Iraq**	21,558	144.1	56,764	163.3	15,824	132.0	46,038	190.9	37,382	136.6	102,802	175.0
**Ireland**	13,990	307.5	23,580	68.6	17,252	386.3	30,797	78.5	31,242	344.7	54,377	74.1
**Israel**	15,692	244.1	26,571	69.3	14,746	250.3	27,176	84.3	30,438	245.8	53,747	76.6
**Italy**	204,092	264.1	230,263	12.8	232,150	312.1	299,518	29.0	436,242	284.5	529,781	21.4
**Jamaica**	3,787	197.4	5,397	42.5	3,713	202.5	5,352	44.1	7,500	199.6	10,749	43.3
**Japan**	424,622	234.2	430,343	1.4	580,535	309.8	628,444	8.3	1,005,157	267.1	1,058,786	5.3
**Jordan**	6,634	157.6	15,465	133.1	5,694	149.9	16,038	181.7	12,328	152.6	31,503	155.5
**Kazakhstan**	19,278	152.2	28,374	47.2	16,947	184.4	27,925	64.8	36,225	162.3	56,299	55.4

© 2024 Bizuayehu HM et al. *JAMA Network Open.*

Country or territory	Females				Males				Both sexes			
	2022 cases	ASIR[a]	2050 cases	Change (%)[b]	2022 cases	ASIR[a]	2050 cases	Change (%)[b]	2022 cases	ASIR[a]	2050 cases	Change (%)[b]
**Kenya**	28,377	168.1	70,925	149.9	16,349	133.1	45,559	178.7	44,726	149.1	116,484	160.4
**North Korea**	32,135	155.0	44,468	38.4	29,398	187.5	53,761	82.9	61,533	166.1	98,228	59.6
**South Korea**	109,656	228.5	145,793	33.0	128,045	252.5	218,246	70.4	237,701	234.7	364,039	53.2
**Kuwait**	2,227	139.1	7,306	228.1	2,120	97.8	11,744	454.0	4,347	111.4	19,049	338.2
**Kyrgyzstan**	3,895	117.6	7,407	90.2	3,371	143.3	6,929	105.6	7,266	125.8	14,336	97.3
**Lao People's Democratic Republic**	4,522	143.9	9,496	110.0	4,579	167.9	10,403	127.2	9,101	154.5	19,899	118.7
**Latvia**	5,550	249.4	4,647	-16.3	5,908	364.0	6,420	8.7	11,458	288.9	11,067	-3.4
**Lebanon**	6,441	170.2	7,873	22.2	6,593	170.8	8,002	21.4	13,034	168.8	15,875	21.8
**Lesotho**	1,302	130.4	2,022	55.3	725	108.1	1,263	74.2	2,027	115.5	3,284	62.0
**Liberia**	2,325	128.8	5,102	119.4	1,548	102.9	3,528	127.9	3,873	115.2	8,630	122.8
**Libya**	4,101	120.1	8,722	112.7	4,070	150.8	10,937	168.7	8,171	132.3	19,658	140.6
**Lithuania**	7,168	224.0	6,271	-12.5	9,245	391.3	10,297	11.4	16,413	287.9	16,567	0.9
**Luxembourg**	1,698	267.2	3,076	81.2	1,742	285.8	3,689	111.8	3,440	273.3	6,764	96.6
**Madagascar**	12,797	130.7	32,583	154.6	8,500	104.9	22,840	168.7	21,297	117.4	55,422	160.2
**Malawi**	12,431	184.9	32,557	161.9	7,415	135.0	18,307	146.9	19,846	159.5	50,864	156.3
**Malaysia**	26,758	148.0	54,576	104.0	24,892	137.9	55,279	122.1	51,650	142.1	109,855	112.7
**Maldives**	231	111.3	495	114.3	248	107.1	950	283.1	479	105.3	1,445	201.7
**Mali**	9,597	164.5	25,974	170.7	5,554	113.9	15,921	186.7	15,151	139.9	41,894	176.5
**Malta**	1,326	271.6	1,928	45.4	1,529	299.8	2,768	81.0	2,855	282.5	4,696	64.5
**Mauritania**	2,008	120.0	4,810	139.5	1,266	91.4	2,935	131.8	3,274	105.3	7,745	136.6
**Mauritius**	1,619	152.6	2,187	35.1	1,269	130.0	1,913	50.8	2,888	140.0	4,101	42.0
**Mexico**	111,200	141.8	199,645	79.5	95,954	141.3	193,239	101.4	207,154	140.9	392,884	89.7
**Moldova**	6,831	189.4	5,923	-13.3	7,985	295.3	7,244	-9.3	14,816	232.2	13,167	-11.1
**Mongolia**	3,200	202.0	8,354	161.1	3,499	280.4	8,533	143.9	6,699	235.8	16,887	152.1
**Montenegro**	1,324	221.1	1,619	22.3	1,415	261.9	1,795	26.9	2,739	238.0	3,414	24.6
**Morocco**	32,872	150.3	52,665	60.2	30,737	150.8	58,348	89.8	63,609	149.8	111,013	74.5
**Mozambique**	16,359	141.4	39,183	139.5	10,219	115.4	26,315	157.5	26,578	128.8	65,498	146.4
**Myanmar**	41,781	129.8	62,863	50.5	35,822	147.2	63,982	78.6	77,603	135.5	126,844	63.5
**Namibia**	1,913	183.1	3,832	100.3	1,540	221.4	3,340	116.9	3,453	193.5	7,172	107.7
**Nepal**	12,216	80.7	23,893	95.6	9,792	81.6	18,364	87.5	22,008	81.6	42,258	92.0
**New Caledonia**	518	256.9	971	87.5	631	325.3	1,294	105.1	1,149	287.6	2,265	97.1
**New Zealand**	17,595	386.3	27,386	55.7	20,562	473.4	33,001	60.5	38,157	427.3	60,387	58.3
**Nicaragua**	4,646	133.7	9,810	111.2	3,763	136.4	9,062	140.8	8,409	133.5	18,872	124.4
**Niger**	6,683	92.8	19,642	193.9	4,910	74.2	14,370	192.7	11,593	83.7	34,012	193.4
**Nigeria**	79,667	133.3	183,243	130.0	48,096	93.9	117,699	144.7	127,763	113.6	300,943	135.6
**North Macedonia**	3,304	180.5	4,180	26.5	4,259	239.8	6,047	42.0	7,563	206.9	10,227	35.2
**Norway**	18,572	337.6	27,421	47.7	21,733	383.6	35,438	63.1	40,305	357.9	62,859	56.0
**Oman**	1,572	107.0	3,656	132.6	2,473	111.5	6,072	145.5	4,045	105.3	9,728	140.5
**Pakistan**	98,180	111.8	214,844	118.8	87,568	99.5	170,221	94.4	185,748	105.6	385,064	107.3
**Panama**	4,197	154.3	8,082	92.6	4,156	158.3	9,123	119.5	8,353	154.9	17,205	106.0
**Papua New Guinea**	6,801	197.8	16,661	145.0	5,389	181.7	13,092	142.9	12,190	188.7	29,753	144.1
**Paraguay**	6,817	187.6	12,390	81.8	6,966	199.8	12,498	79.4	13,783	192.2	24,888	80.6
**Peru**	39,103	185.7	72,588	85.6	33,724	164.6	66,645	97.6	72,827	173.8	139,233	91.2
**Philippines**	105,912	192.5	203,946	92.6	83,064	184.6	190,479	129.3	188,976	185.4	394,426	108.7
**Poland**	99,535	233.6	118,508	19.1	109,365	307.0	152,877	39.8	208,900	262.8	271,385	29.9
**Portugal**	31,702	258.5	35,911	13.3	37,865	342.7	45,560	20.3	69,567	294.6	81,470	17.1
**Puerto Rico**	6,292	199.7	7,885	25.3	7,486	266.6	9,789	30.8	13,778	226.6	17,674	28.3

© 2024 Bizuayehu HM et al. *JAMA Network Open.*

Country or territory	Females				Males				Both sexes			
	2022 cases	ASIR[a]	2050 cases	Change (%)[b]	2022 cases	ASIR[a]	2050 cases	Change (%)[b]	2022 cases	ASIR[a]	2050 cases	Change (%)[b]
**Qatar**	640	119.7	1,564	144.4	1,093	70.7	2,538	132.2	1,733	82.4	4,101	136.6
**Romania**	47,350	238.1	49,957	5.5	57,311	330.8	68,357	19.3	104,661	276.5	118,314	13.1
**Russian Federation**	334,064	230.8	348,958	4.5	301,496	288.5	387,603	28.6	635,560	248.1	736,561	15.9
**Rwanda**	3,835	79.7	9,199	139.9	3,287	91.7	8,361	154.4	7,122	83.2	17,560	146.6
**Saint Lucia**	183	136.6	274	49.7	265	199.3	392	47.9	448	166.8	667	48.9
**Samoa**	209	246.3	403	92.8	191	242.1	376	96.9	400	240.0	779	94.8
**Sao Tome and Principe**	67	90.3	150	123.9	77	124.5	171	122.1	144	104.9	321	122.9
**Saudi Arabia**	13,368	100.4	33,839	153.1	14,745	81.0	48,116	226.3	28,113	87.1	81,954	191.5
**Senegal**	7,571	126.9	19,317	155.1	4,270	99.0	12,161	184.8	11,841	113.9	31,477	165.8
**Serbia**	19,129	219.6	16,626	-13.1	22,910	289.9	21,292	-7.1	42,039	250.4	37,918	-9.8
**Sierra Leone**	1,044	34.7	2,409	130.8	874	39.3	2,332	166.8	1,918	35.9	4,741	147.2
**Singapore**	11,908	231.0	21,440	80.1	13,342	235.9	28,576	114.2	25,250	231.1	50,016	98.1
**Slovakia**	14,215	238.0	18,340	29.0	16,698	346.8	25,358	51.9	30,913	283.3	43,697	41.4
**Slovenia**	6,564	275.5	7,643	16.4	7,838	343.7	10,858	38.5	14,402	305.1	18,502	28.5
**Solomon Islands**	405	154.4	905	123.5	279	114.8	605	116.9	684	134.8	1,510	120.8
**Somalia**	6,683	145.9	17,701	164.9	3,998	95.4	9,744	143.7	10,681	121.0	27,445	157.0
**South Africa**	58,942	190.4	107,020	81.6	52,379	232.4	109,269	108.6	111,321	203.4	216,289	94.3
**South Sudan**	4,017	109.7	8,035	100.0	2,857	89.8	5,439	90.4	6,874	99.4	13,473	96.0
**Spain**	120,319	238.0	151,470	25.9	158,410	319.9	224,869	42.0	278,729	274.6	376,339	35.0
**Sri Lanka**	17,510	106.0	24,802	41.6	15,733	110.6	23,454	49.1	33,243	106.9	48,256	45.2
**Sudan**	17,260	109.7	41,507	140.5	11,326	80.6	26,709	135.8	28,586	95.6	68,216	138.6
**Suriname**	548	154.8	972	77.4	571	195.0	1,124	96.9	1,119	169.5	2,096	87.3
**Sweden**	31,392	289.1	41,665	32.7	37,869	334.8	54,347	43.5	69,261	310.0	96,013	38.6
**Switzerland**	26,334	260.2	38,205	45.1	31,996	329.9	50,808	58.8	58,330	292.7	89,013	52.6
**Syrian Arab Republic**	11,747	136.9	35,394	201.3	10,179	142.7	31,323	207.7	21,926	138.6	66,717	204.3
**Tajikistan**	3,518	92.0	7,921	125.2	2,949	92.7	7,054	139.2	6,467	91.7	14,975	131.6
**Tanzania**	26,716	151.1	79,471	197.5	18,215	132.8	56,172	208.4	44,931	140.1	135,642	201.9
**Thailand**	93,208	150.6	138,371	48.5	90,333	161.6	148,624	64.5	183,541	154.4	286,995	56.4
**The Netherlands**	62,749	336.2	76,934	22.6	69,570	350.8	92,925	33.6	132,319	341.4	169,859	28.4
**The Republic of the Gambia**	676	83.1	1,816	168.6	520	74.7	1,433	175.6	1,196	79.2	3,249	171.7
**Timor-Leste**	455	89.6	917	101.5	373	80.8	755	102.4	828	84.5	1,672	101.9
**Togo**	3,095	111.1	7,272	135.0	2,396	105.4	6,250	160.9	5,491	107.5	13,522	146.3
**Trinidad and Tobago**	1,962	182.1	3,182	62.2	1,969	195.5	3,316	68.4	3,931	186.7	6,498	65.3
**Tunisia**	9,110	115.3	15,920	74.8	11,441	159.4	21,564	88.5	20,551	135.4	37,484	82.4
**Türkiye**	107,537	192.2	177,149	64.7	132,476	273.7	281,160	112.2	240,013	225.9	458,309	91.0
**Turkmenistan**	3,760	121.4	7,020	86.7	3,047	128.8	6,278	106.0	6,807	122.7	13,298	95.4
**Uganda**	20,509	157.7	55,462	170.4	15,459	156.2	42,249	173.3	35,968	154.4	97,711	171.7
**Ukraine**	79,414	182.1	72,570	-8.6	75,825	236.4	86,552	14.2	155,239	199.9	159,122	2.5
**United Arab Emirates**	2,919	157.8	9,222	215.9	2,607	86.4	10,283	294.4	5,526	105.1	19,504	253.0
**United Kingdom**	212,709	292.5	278,770	31.1	242,245	327.7	345,812	42.8	454,954	307.8	624,582	37.3
**United States of America**	1,096,291	341.7	1,523,769	39.0	1,283,898	401.7	1,986,528	54.7	2,380,189	367.0	3,510,297	47.5
**Uruguay**	8,238	253.2	10,209	23.9	8,579	322.3	12,441	45.0	16,817	279.9	22,650	34.7
**Uzbekistan**	19,865	114.6	35,530	78.9	16,035	112.9	32,102	100.2	35,900	112.7	67,631	88.4
**Vanuatu**	145	121.7	343	136.6	105	94.0	232	121.0	250	108.2	576	130.4
**Venezuela**	31,573	174.5	55,597	76.1	31,374	201.6	55,950	78.3	62,947	184.8	111,547	77.2

© 2024 Bizuayehu HM et al. *JAMA Network Open.*

Country or territory	Females				Males				Both sexes			
	**2022 cases**	**ASIR[a]**	**2050 cases**	**Change (%)[b]**	**2022 cases**	**ASIR[a]**	**2050 cases**	**Change (%)[b]**	**2022 cases**	**ASIR[a]**	**2050 cases**	**Change (%)[b]**
**Vietnam**	85,122	132.3	135,919	59.7	95,358	177.1	174,053	82.5	180,480	150.8	309,972	71.8
**Yemen**	9,312	89.2	25,058	169.1	7,213	77.8	19,135	165.3	16,525	83.1	44,193	167.4
**Zambia**	8,863	164.8	26,069	194.1	6,433	162.6	20,207	214.1	15,296	159.5	46,276	202.5
**Zimbabwe**	11,220	220.5	28,085	150.3	6,505	202.5	17,022	161.7	17,725	208.0	45,106	154.5

[a] The estimates for ASIR are per 100,000 persons and were adjusted using the World Standard Population.
[b] Negative percentage change values indicate a decrease in cancer cases between 2022 and 2050.

ASIR=Age-Standardised Incidence Rate

© 2024 Bizuayehu HM et al. *JAMA Network Open.*

eTable 5. Distribution of Cancer Deaths and Mortality Rate in 2022 and the Projected Deaths and Percentage of Change by 2050 in Each Country or Territory Sorted by Alphabetical Order

Country or territory	Females				Males				Both sexes			
	2022 Deaths	ASMR[a]	2050 Deaths	Change (%)[b]	2022 Deaths	ASMR[a]	2050 Deaths	Change (%)[b]	2022 Deaths	ASMR[a]	2050 Deaths	Change (%)[b]
**Afghanistan**	8,561	77.5	23,413	173.5	8,362	81.0	20,678	147.3	16,923	78.7	44,091	160.5
**Albania**	1,906	66.3	2,918	53.1	3,049	116.6	4,088	34.1	4,955	90.5	7,007	41.4
**Algeria**	16,969	73.1	37,300	119.8	18,809	82.7	47,346	151.7	35,778	77.7	84,646	136.6
**Angola**	8,543	90.7	24,498	186.8	6,998	91.5	20,317	190.3	15,541	90.0	44,814	188.4
**Argentina**	33,663	88.7	54,855	63.0	36,588	123.6	69,828	90.9	70,251	102.7	124,683	77.5
**Armenia**	2,617	87.0	3,985	52.3	3,244	160.3	3,937	21.4	5,861	116.7	7,921	35.2
**Australia**	22,516	71.2	42,084	86.9	29,368	100.2	56,664	92.9	51,884	84.6	98,748	90.3
**Austria**	10,214	76.7	14,695	43.9	12,167	109.5	19,715	62.0	22,381	91.3	34,410	53.8
**Azerbaijan**	4,713	71.0	8,655	83.6	6,601	122.8	12,740	93.0	11,314	93.2	21,395	89.1
**Bahamas**	286	103.3	672	135.0	266	119.2	656	146.6	552	109.2	1,328	140.6
**Bahrain**	295	62.8	977	231.2	312	59.1	1,547	395.8	607	58.6	2,524	315.8
**Bangladesh**	48,007	60.9	111,606	132.5	68,591	87.8	140,803	105.3	116,598	74.7	252,409	116.5
**Barbados**	357	104.5	457	28.0	381	124.9	493	29.4	738	113.3	950	28.7
**Belarus**	9,080	79.4	10,948	20.6	13,564	192.7	17,943	32.3	22,644	123.5	28,891	27.6
**Belgium**	12,960	76.6	18,026	39.1	16,045	111.7	25,424	58.5	29,005	92.4	43,450	49.8
**Belize**	105	57.2	284	170.5	106	62.2	273	157.6	211	59.9	557	164.0
**Benin**	2,599	67.6	6,447	148.1	2,470	79.4	6,667	169.9	5,069	71.6	13,114	158.7
**Bhutan**	200	61.2	510	155.0	280	72.4	663	136.8	480	67.2	1,173	144.4
**Bolivia**	6,057	91.2	10,408	71.8	4,958	79.5	7,546	52.2	11,015	85.1	17,955	63.0
**Bosnia Herzegovina**	3,539	87.9	3,996	12.9	5,051	151.2	6,373	26.2	8,590	116.1	10,368	20.7
**Botswana**	725	64.8	1,520	109.7	552	70.2	1,416	156.5	1,277	66.1	2,936	129.9
**Brazil**	132,133	79.4	251,938	90.7	146,702	107.7	301,873	105.8	278,835	91.3	553,811	98.6
**Brunei Darussalam**	235	104.2	696	196.2	229	105.6	675	194.8	464	104.6	1,370	195.3
**Bulgaria**	8,031	88.0	7,699	-4.1	10,763	148.8	11,698	8.7	18,794	114.2	19,397	3.2
**Burkina Faso**	6,654	106.7	17,411	161.7	4,344	91.1	12,406	185.6	10,998	98.5	29,817	171.1
**Burundi**	3,329	103.4	9,774	193.6	2,431	95.3	7,465	207.1	5,760	98.7	17,239	199.3
**Cambodia**	6,649	83.4	14,669	120.6	7,150	121.9	18,384	157.1	13,799	99.3	33,053	139.5
**Cameroon**	7,486	95.8	19,538	161.0	5,312	79.7	14,346	170.1	12,798	87.3	33,884	164.8
**Canada**	47,751	85.0	89,315	87.0	52,714	110.4	106,533	102.1	100,465	96.4	195,848	94.9
**Cape Verde**	136	48.8	372	173.5	149	75.7	513	244.3	285	58.3	885	210.5
**Central African Republic**	1,202	85.2	2,768	130.3	821	76.5	1,976	140.7	2,023	79.9	4,744	134.5
**Chad**	3,320	71.7	8,847	166.5	3,937	104.4	10,262	160.7	7,257	86.2	19,109	163.3
**Chile**	14,543	77.6	28,102	93.2	16,897	107.8	40,016	136.8	31,440	90.1	68,118	116.7
**China**	944,888	67.8	1,830,491	93.7	1,629,288	127.5	2,990,715	83.6	2,574,176	96.5	4,821,206	87.3
**Colombia**	28,903	76.9	60,023	107.7	27,816	87.6	61,915	122.6	56,719	81.4	121,938	115.0
**Comoros**	251	85.2	564	124.7	167	65.9	415	148.5	418	75.3	978	134.0
**Democratic Republic of Congo**	20,748	78.6	54,128	160.9	16,827	75.2	42,800	154.4	37,575	76.2	96,927	158.0
**Republic of Congo**	917	50.3	2,189	138.7	815	59.2	2,226	173.1	1,732	52.9	4,415	154.9
**Costa Rica**	2,826	67.6	6,082	115.2	3,246	84.9	7,448	129.5	6,072	75.3	13,530	122.8
**Côte d'Ivoire**	7,459	96.8	19,423	160.4	6,684	90.5	16,068	140.4	14,143	93.6	35,491	150.9
**Croatia**	5,725	91.9	5,967	4.2	8,129	172.0	10,536	29.6	13,854	125.7	16,504	19.1

© 2024 Bizuayehu HM et al. *JAMA Network Open.*

Country or territory	Females				Males				Both sexes			
	2022 Deaths	ASMR[a]	2050 Deaths	Change (%)[b]	2022 Deaths	ASMR[a]	2050 Deaths	Change (%)[b]	2022 Deaths	ASMR[a]	2050 Deaths	Change (%)[b]
**Cuba**	11,870	91.2	17,374	46.4	15,854	133.1	24,053	51.7	27,724	110.2	41,427	49.4
**Cyprus**	1,263	92.6	2,509	98.7	1,574	134.8	3,505	122.7	2,837	111.5	6,014	112.0
**Czechia**	12,583	82.8	14,641	16.4	15,488	129.9	20,768	34.1	28,071	103.8	35,409	26.1
**Denmark**	8,080	95.9	11,132	37.8	9,124	119.7	13,814	51.4	17,204	106.5	24,946	45.0
**Djibouti**	335	78.1	780	132.8	237	56.3	505	113.1	572	66.7	1,285	124.7
**Dominican Republic**	5,568	85.8	11,123	99.8	6,176	100.4	12,915	109.1	11,744	92.0	24,037	104.7
**Ecuador**	8,500	77.1	19,787	132.8	7,658	75.9	19,022	148.4	16,158	76.0	38,809	140.2
**Egypt**	43,583	92.2	98,214	125.4	51,692	127.4	118,797	129.8	95,275	107.7	217,010	127.8
**El Salvador**	3,002	64.6	5,240	74.6	2,290	65.1	3,632	58.6	5,292	64.7	8,873	67.7
**Equatorial Guinea**	310	82.6	1,056	240.7	279	64.7	923	230.8	589	72.1	1,979	236.0
**Eritrea**	1,072	84.9	2,474	130.8	652	59.0	1,462	124.2	1,724	72.5	3,936	128.3
**Estonia**	1,885	82.9	2,043	8.4	2,201	164.7	3,259	48.1	4,086	114.4	5,301	29.7
**Eswatini**	466	110.9	1,034	121.9	231	75.9	538	132.9	697	88.8	1,573	125.7
**Ethiopia**	34,967	91.4	93,424	167.2	19,731	56.5	50,872	157.8	54,698	74.5	144,297	163.8
**Fiji**	541	117.0	900	66.4	324	80.3	527	62.7	865	98.5	1,427	65.0
**Finland**	6,173	67.8	7,598	23.1	7,272	95.2	10,083	38.7	13,445	79.8	17,681	31.5
**France (metropolitan)**	83,691	82.8	115,928	38.5	106,921	135.7	152,614	42.7	190,612	106.7	268,542	40.9
**France, Guadeloupe**	419	73.5	612	46.1	533	119.4	689	29.3	952	93.0	1,301	36.7
**France, La Réunion**	625	70.4	1,293	106.9	955	125.1	1,871	95.9	1,580	95.2	3,164	100.3
**France, Martinique**	427	68.5	592	38.6	459	93.3	552	20.3	886	79.3	1,144	29.1
**French Guyana**	95	63.6	259	172.6	138	109.7	447	223.9	233	82.6	706	203.0
**French Polynesia**	185	94.2	451	143.8	296	152.0	771	160.5	481	122.3	1,222	154.1
**Gabon**	614	76.8	1,508	145.6	480	62.8	1,184	146.7	1,094	69.5	2,693	146.2
**Gaza Strip and West Bank**	1,335	81.5	4,120	208.6	1,676	117.5	5,138	206.6	3,011	98.0	9,257	207.4
**Georgia**	3,823	84.5	4,127	8.0	4,636	151.7	4,793	3.4	8,459	111.7	8,920	5.5
**Germany**	115,344	83.9	143,698	24.6	137,826	118.7	189,040	37.2	253,170	99.7	332,738	31.4
**Ghana**	9,856	87.8	24,149	145.0	8,088	77.6	19,309	138.7	17,944	82.6	43,458	142.2
**Greece**	13,265	76.4	16,885	27.3	19,120	129.7	24,440	27.8	32,385	100.6	41,325	27.6
**Guam**	114	80.9	216	89.5	153	125.8	303	98.0	267	101.1	519	94.4
**Guatemala**	5,825	73.6	13,738	135.9	4,544	68.4	10,908	140.1	10,369	71.1	24,647	137.7
**Guinea**	3,931	90.3	9,364	138.2	2,432	85.0	7,540	210.0	6,363	87.5	16,903	165.7
**Guinea-Bissau**	526	84.3	1,337	154.2	323	67.9	917	183.9	849	76.5	2,254	165.5
**Guyana**	341	76.6	552	61.9	271	64.7	410	51.3	612	70.2	962	57.2
**Haiti**	4,419	82.6	7,988	80.8	4,595	106.2	7,918	72.3	9,014	92.2	15,906	76.5
**Honduras**	3,647	82.6	8,475	132.4	3,364	85.5	7,933	135.8	7,011	83.3	16,408	134.0
**Hungary**	15,127	112.2	16,633	10.0	17,355	187.9	22,979	32.4	32,482	143.7	39,612	22.0
**Iceland**	320	77.0	572	78.8	349	90.8	709	103.2	669	83.2	1,281	91.5
**India**	446,772	62.6	884,299	97.9	470,055	66.5	947,302	101.5	916,827	64.4	1,831,601	99.8
**Indonesia**	114,248	73.9	204,247	78.8	128,740	93.9	242,459	88.3	242,988	82.5	446,706	83.8
**Iran**	36,534	81.4	114,246	212.7	50,713	110.8	137,898	171.9	87,247	96.5	252,145	189.0
**Iraq**	11,133	79.5	31,941	186.9	10,403	91.9	31,756	205.3	21,536	84.4	63,697	195.8
**Ireland**	4,975	90.0	9,819	97.4	5,463	109.1	11,268	106.3	10,438	98.7	21,086	102.0
**Israel**	6,355	75.5	11,961	88.2	6,500	92.9	13,538	108.3	12,855	83.2	25,499	98.4
**Italy**	89,847	79.0	118,514	31.9	103,859	113.3	153,213	47.5	193,706	94.2	271,728	40.3
**Jamaica**	2,209	110.6	3,410	54.4	2,423	122.9	3,487	43.9	4,632	116.6	6,897	48.9
**Japan**	185,455	61.3	212,355	14.5	240,823	100.7	290,614	20.7	426,278	78.6	502,969	18.0

© 2024 Bizuayehu HM et al. *JAMA Network Open.*

Country or territory	Females				Males				Both sexes			
	2022 Deaths	ASMR[a]	2050 Deaths	Change (%)[b]	2022 Deaths	ASMR[a]	2050 Deaths	Change (%)[b]	2022 Deaths	ASMR[a]	2050 Deaths	Change (%)[b]
**Jordan**	3,005	75.5	8,038	167.5	3,453	94.5	10,488	203.7	6,458	84.3	18,526	186.9
**Kazakhstan**	9,321	71.6	14,218	52.5	11,365	123.9	18,835	65.7	20,686	92.1	33,054	59.8
**Kenya**	18,003	112.8	47,275	162.6	11,314	98.4	33,339	194.7	29,317	104.0	80,614	175.0
**North Korea**	20,404	93.9	30,378	48.9	22,385	143.2	42,224	88.6	42,789	113.9	72,602	69.7
**South Korea**	36,556	51.0	81,063	121.8	61,059	110.6	137,640	125.4	97,615	77.0	218,703	124.1
**Kuwait**	870	65.9	4,536	421.4	1,098	58.0	8,147	642.0	1,968	60.2	12,682	544.4
**Kyrgyzstan**	2,218	68.0	4,506	103.2	2,454	105.9	5,151	109.9	4,672	82.8	9,657	106.7
**Lao People's Democratic Republic**	2,671	89.4	6,174	131.2	3,544	132.9	8,291	133.9	6,215	109.9	14,464	132.7
**Latvia**	2,712	89.5	2,457	-9.4	3,160	178.5	3,669	16.1	5,872	122.9	6,126	4.3
**Lebanon**	3,357	85.8	4,975	48.2	3,950	101.1	5,457	38.2	7,307	92.3	10,432	42.8
**Lesotho**	904	90.9	1,396	54.4	507	78.0	883	74.2	1,411	82.0	2,280	61.6
**Liberia**	1,593	91.9	3,547	122.7	1,137	76.6	2,607	129.3	2,730	84.0	6,154	125.4
**Libya**	2,396	74.4	6,367	165.7	2,920	113.6	8,783	200.8	5,316	91.1	15,151	185.0
**Lithuania**	3,710	88.7	3,532	-4.8	4,639	181.6	5,555	19.8	8,349	125.0	9,087	8.8
**Luxembourg**	517	68.9	1,033	99.8	576	87.4	1,299	125.5	1,093	77.3	2,332	113.4
**Madagascar**	8,222	89.2	22,001	167.6	6,048	78.9	16,905	179.5	14,270	83.6	38,906	172.6
**Malawi**	8,748	137.0	23,555	169.3	5,231	98.8	13,136	151.1	13,979	117.9	36,691	162.5
**Malaysia**	14,653	79.9	34,986	138.8	16,980	93.1	40,466	138.3	31,633	86.3	75,453	138.5
**Maldives**	91	48.8	275	202.2	150	68.5	666	344.0	241	58.8	941	290.5
**Mali**	6,469	115.1	17,565	171.5	4,209	89.1	12,166	189.1	10,678	102.4	29,731	178.4
**Malta**	466	69.7	744	59.7	570	96.1	1,113	95.3	1,036	81.6	1,858	79.3
**Mauritania**	1,326	82.6	3,247	144.9	908	67.4	2,128	134.4	2,234	74.8	5,375	140.6
**Mauritius**	754	63.9	1,171	55.3	715	71.5	1,200	67.8	1,469	66.6	2,371	61.4
**Mexico**	49,795	61.4	102,953	106.8	46,415	66.5	101,408	118.5	96,210	63.5	204,361	112.4
**Moldova**	3,301	84.7	3,023	-8.4	4,847	177.5	4,489	-7.4	8,148	122.8	7,511	-7.8
**Mongolia**	2,210	145.5	6,930	213.6	2,719	227.5	7,109	161.5	4,929	181.5	14,038	184.8
**Montenegro**	688	98.8	944	37.2	847	149.7	1,137	34.2	1,535	121.4	2,081	35.6
**Morocco**	15,792	70.5	29,507	86.9	21,155	105.0	43,776	106.9	36,947	85.9	73,283	98.4
**Mozambique**	11,629	104.8	28,021	141.0	7,391	87.3	19,126	158.8	19,020	96.3	47,147	147.9
**Myanmar**	27,323	85.1	44,654	63.4	27,518	114.9	50,981	85.3	54,841	97.1	95,634	74.4
**Namibia**	1,043	103.4	2,178	108.8	897	134.0	1,987	121.5	1,940	113.0	4,165	114.7
**Nepal**	7,768	52.5	16,144	107.8	6,936	58.1	13,157	89.7	14,704	55.3	29,302	99.3
**New Caledonia**	194	90.8	441	127.3	273	139.8	640	134.4	467	113.2	1,081	131.5
**New Zealand**	5,236	90.0	9,595	83.3	6,065	112.6	11,884	95.9	11,301	100.2	21,479	90.1
**Nicaragua**	2,516	72.2	5,640	124.2	2,306	84.0	5,649	145.0	4,822	76.8	11,289	134.1
**Niger**	4,758	69.1	13,921	192.6	4,048	63.3	11,907	194.2	8,806	66.1	25,828	193.3
**Nigeria**	46,637	81.4	108,873	133.5	32,905	68.4	82,600	151.0	79,542	74.6	191,474	140.7
**North Macedonia**	1,757	86.2	2,484	41.4	2,607	143.1	3,888	49.1	4,364	112.5	6,372	46.0
**Norway**	6,538	83.5	11,219	71.6	6,855	102.1	13,411	95.6	13,393	91.8	24,629	83.9
**Oman**	751	57.4	2,087	177.9	1,510	75.2	4,207	178.6	2,261	66.3	6,294	178.4
**Pakistan**	59,697	70.8	135,271	126.6	58,934	68.8	116,245	97.3	118,631	69.8	251,516	112.0
**Panama**	1,807	61.9	3,923	117.1	1,963	71.0	4,623	135.5	3,770	65.7	8,546	126.7
**Papua New Guinea**	3,926	122.2	10,429	165.6	3,274	118.6	8,299	153.5	7,200	119.8	18,728	160.1
**Paraguay**	3,049	81.8	6,152	101.8	3,532	99.8	6,594	86.7	6,581	89.9	12,746	93.7
**Peru**	19,144	86.2	38,702	102.2	16,790	79.8	34,456	105.2	35,934	82.4	73,158	103.6
**Philippines**	56,506	102.0	117,624	108.2	56,863	129.3	136,462	140.0	113,369	112.9	254,086	124.1

© 2024 Bizuayehu HM et al. *JAMA Network Open.*

Country or territory	Females				Males				Both sexes			
	2022 Deaths	ASMR[a]	2050 Deaths	Change (%)[b]	2022 Deaths	ASMR[a]	2050 Deaths	Change (%)[b]	2022 Deaths	ASMR[a]	2050 Deaths	Change (%)[b]
**Poland**	54,250	104.3	69,634	28.4	65,742	172.8	98,079	49.2	119,992	133.1	167,713	39.8
**Portugal**	14,252	77.5	18,968	33.1	19,510	148.8	26,232	34.5	33,762	109.0	45,200	33.9
**Puerto Rico**	2,584	59.9	3,850	49.0	3,212	96.1	4,878	51.9	5,796	75.0	8,728	50.6
**Qatar**	255	61.8	867	240.0	527	40.2	1,514	187.3	782	46.2	2,381	204.5
**Romania**	23,386	96.1	26,802	14.6	32,830	179.0	40,866	24.5	56,216	132.5	67,668	20.4
**Russian Federation**	147,557	84.9	168,880	14.5	164,172	153.0	221,454	34.9	311,729	110.5	390,335	25.2
**Rwanda**	2,563	55.8	6,394	149.5	2,324	67.0	6,002	158.3	4,887	59.4	12,396	153.7
**Saint Lucia**	91	63.4	159	74.7	132	89.5	197	49.2	223	76.1	356	59.6
**Samoa**	97	113.7	196	102.1	111	144.7	235	111.7	208	126.3	431	107.2
**Sao Tome and Principe**	42	60.1	99	135.7	48	78.6	108	125.0	90	68.0	207	130.0
**Saudi Arabia**	5,349	45.0	17,294	223.3	8,050	48.4	33,027	310.3	13,399	46.2	50,321	275.6
**Senegal**	5,023	88.1	13,333	165.4	3,111	74.2	8,996	189.2	8,134	81.6	22,329	174.5
**Serbia**	10,311	101.9	9,870	-4.3	13,570	161.0	13,261	-2.3	23,881	128.4	23,132	-3.1
**Sierra Leone**	701	24.7	1,673	138.7	633	27.7	1,655	161.5	1,334	25.5	3,328	149.5
**Singapore**	5,618	92.9	14,280	154.2	7,659	131.4	21,438	179.9	13,277	110.8	35,718	169.0
**Slovakia**	6,773	95.9	9,716	43.5	8,393	166.9	14,101	68.0	15,166	125.7	23,817	57.0
**Slovenia**	3,022	92.6	4,020	33.0	3,876	147.3	6,325	63.2	6,898	116.7	10,345	50.0
**Solomon Islands**	199	78.4	461	131.7	166	70.8	366	120.5	365	74.6	827	126.6
**Somalia**	4,934	112.6	13,024	164.0	3,104	78.1	7,546	143.1	8,038	95.5	20,570	155.9
**South Africa**	33,894	110.6	70,503	108.0	30,653	146.3	69,915	128.1	64,547	122.5	140,417	117.5
**South Sudan**	2,938	83.3	5,930	101.8	2,143	69.8	4,107	91.7	5,081	76.3	10,037	97.5
**Spain**	46,148	65.3	69,697	51.0	69,442	117.2	112,880	62.6	115,590	88.8	182,578	58.0
**Sri Lanka**	9,325	52.6	14,398	54.4	9,820	67.8	15,041	53.2	19,145	59.0	29,440	53.8
**Sudan**	10,503	69.7	25,988	147.4	8,001	58.7	18,938	136.7	18,504	64.4	44,926	142.8
**Suriname**	286	76.1	582	103.5	340	114.8	722	112.4	626	91.5	1,304	108.3
**Sweden**	12,361	80.0	17,809	44.1	13,208	92.4	21,664	64.0	25,569	85.2	39,474	54.4
**Switzerland**	8,828	67.6	14,318	62.2	10,592	92.3	19,364	82.8	19,420	78.8	33,681	73.4
**Syrian Arab Republic**	6,631	80.5	21,698	227.2	7,217	103.7	22,843	216.5	13,848	91.0	44,541	221.6
**Tajikistan**	2,076	58.3	5,170	149.0	2,149	70.7	5,369	149.8	4,225	63.9	10,539	149.4
**Tanzania**	16,947	100.1	52,007	206.9	12,796	97.2	40,321	215.1	29,743	97.1	92,327	210.4
**Thailand**	53,729	78.2	96,388	79.4	65,100	112.4	116,371	78.8	118,829	93.4	212,758	79.1
**The Netherlands**	22,618	92.3	31,709	40.2	27,172	116.7	42,573	56.7	49,790	103.2	74,282	49.2
**The Republic of the Gambia**	459	61.0	1,289	180.8	440	64.4	1,229	179.3	899	62.7	2,518	180.1
**Timor-Leste**	255	53.0	548	114.9	262	59.0	548	109.2	517	55.4	1,096	112.0
**Togo**	1,982	74.4	4,781	141.2	1,623	74.7	4,323	166.4	3,605	74.0	9,104	152.5
**Trinidad and Tobago**	1,052	89.1	1,993	89.5	1,169	109.1	2,306	97.3	2,221	96.8	4,298	93.5
**Tunisia**	4,819	57.5	10,175	111.1	7,761	106.6	15,803	103.6	12,580	80.2	25,978	106.5
**Türkiye**	47,226	75.8	95,914	103.1	82,446	168.3	191,785	132.6	129,672	116.1	287,699	121.9
**Turkmenistan**	2,273	74.8	4,540	99.7	2,183	94.6	4,647	112.9	4,456	82.5	9,188	106.2
**Uganda**	13,921	115.5	38,584	177.2	10,708	116.3	29,965	179.8	24,629	113.9	68,549	178.3
**Ukraine**	36,738	73.7	36,636	-0.3	47,415	144.4	55,731	17.5	84,153	101.1	92,367	9.8
**United Arab Emirates**	1,000	70.3	4,445	344.5	1,283	48.7	5,916	361.1	2,283	54.4	10,361	353.8
**United Kingdom**	84,678	87.6	123,952	46.4	97,129	111.3	155,052	59.6	181,807	98.3	279,004	53.5
**United States of America**	285,120	73.6	437,258	53.4	320,641	93.2	537,573	67.7	605,761	82.3	974,831	60.9
**Uruguay**	4,060	101.4	5,296	30.4	4,920	168.1	7,583	54.1	8,980	128.3	12,879	43.4
**Uzbekistan**	11,328	67.3	22,038	94.5	10,743	77.5	22,314	107.7	22,071	71.5	44,352	101.0

© 2024 Bizuayehu HM et al. *JAMA Network Open.*

Country or territory	Females				Males				Both sexes			
	2022 Deaths	ASMR[a]	2050 Deaths	Change (%)[b]	2022 Deaths	ASMR[a]	2050 Deaths	Change (%)[b]	2022 Deaths	ASMR[a]	2050 Deaths	Change (%)[b]
**Vanuatu**	74	65.2	184	148.7	77	69.8	172	123.4	151	67.8	356	135.8
**Venezuela**	15,391	82.5	29,003	88.4	16,346	104.6	30,434	86.2	31,737	91.3	59,437	87.3
**Vietnam**	48,799	72.7	88,518	81.4	71,385	132.6	135,648	90.0	120,184	99.0	224,165	86.5
**Yemen**	6,339	64.5	17,904	182.4	5,515	63.8	15,355	178.4	11,854	63.7	33,259	180.6
**Zambia**	5,649	113.1	17,257	205.5	4,121	110.7	13,217	220.7	9,770	109.2	30,474	211.9
**Zimbabwe**	7,363	150.9	18,849	156.0	4,376	142.3	11,525	163.4	11,739	144.0	30,375	158.8

[a] The estimates for ASMR are per 100,000 persons and was adjusted using the World Standard Population.
[b] Negative percentage change values indicate a decrease in cancer deaths between 2022 and 2050.

ASMR=Age-Standardised Mortality Rate

© 2024 Bizuayehu HM et al. *JAMA Network Open.*

eTable 6. Cancer Prevalence in 2022 and Over the Past 5 Years (2018-2022) by Country or Territory

Country or territory	Females				Males				Both sexes			
	2022		2018-2022		2022		2018-2022		2022		2018-2022	
	Survivors[a]	Prevalence[b]	Survivors[c]	Prevalence[d]	Survivors[a]	Prevalence[b]	Survivors[c]	Prevalence[d]	Survivors[a]	Prevalence[b]	Survivors[c]	Prevalence[d]
**Afghanistan**	6,779	34.1	27,192	137.0	5,722	27.4	21,035	100.6	12,501	30.7	48,227	118.3
**Albania**	2,490	176.9	9,694	688.6	2,937	201.4	9,978	684.1	5,427	189.3	19,672	686.3
**Algeria**	25,696	114.5	105,310	469.4	19,394	84.6	72,408	316.0	45,090	99.4	177,718	391.9
**Angola**	7,955	45.0	33,150	187.3	5,891	34.0	22,489	129.8	13,846	39.5	55,639	158.8
**Argentina**	52,583	223.3	215,919	916.7	47,020	209.4	180,039	801.7	99,603	216.5	395,958	860.6
**Armenia**	3,060	194.4	11,638	739.3	3,166	226.5	10,713	766.5	6,226	209.5	22,351	752.1
**Australia**	84,551	646.0	350,280	2,676.1	100,806	776.7	406,497	3,131.8	185,357	711	756,777	2,903.0
**Austria**	19,053	415.0	75,374	1,641.9	21,752	486.0	83,302	1,861.1	40,805	450.1	158,676	1,750.1
**Azerbaijan**	6,079	118.0	24,113	468.2	6,331	122.9	22,505	4,37.0	12,410	120.5	46,618	452.6
**Bahamas**	388	188.5	1,595	775.0	379	194.7	1,479	759.6	767	191.5	3,074	767.5
**Bahrain**	637	101.7	2,634	420.3	524	45.3	1,990	172.0	1,161	65.1	4,624	259.2
**Bangladesh**	44,127	53.1	164,393	197.8	54,069	63.8	181,944	214.6	98,196	58.5	346,337	206.3
**Barbados**	415	279.6	1,671	1,125.9	443	317.3	1,763	1,262.8	858	297.9	3,434	1,192.3
**Belarus**	16,563	328.8	68,803	1,365.7	17,937	408.1	68,731	1,563.8	34,500	365.7	137,534	1,458.0
**Belgium**	31,766	540.5	128,230	2,181.9	35,494	612.9	135,939	2,347.3	67,260	576.4	264,169	2,264.0
**Belize**	163	78.6	601	289.7	131	64.0	423	206.6	294	71.3	1,024	248.4
**Benin**	2,196	34.3	8,686	135.8	1,822	28.5	6,313	98.8	4,018	31.4	14,999	117.3
**Bhutan**	196	53.2	613	166.4	235	56.0	633	150.9	431	54.7	1,246	158.1
**Bolivia**	6,572	109.9	25,683	429.6	4,749	79.0	16,332	271.5	11,321	94.4	42,015	350.3
**Bosnia Herzegovina**	4,552	274.4	17,555	1,058.4	5,119	321.8	17,760	1,116.5	9,671	297.6	35,315	1,086.9
**Botswana**	1,034	82.3	4,173	332.2	674	56.9	2,471	208.5	1,708	70.0	6,644	272.2
**Brazil**	218,052	198.9	862,997	787.3	214,911	203.3	771,444	729.6	432,963	201.1	1,634,441	759.0
**Brunei Darussalam**	442	206.0	1,765	822.5	309	133.9	1,087	470.9	751	168.6	2,852	640.3
**Bulgaria**	11,364	322.7	45,591	1,294.5	12,220	367.8	45,440	1,367.6	23,584	344.6	91,031	1,330.0
**Burkina Faso**	4,360	39.5	16,742	1,51.6	2,678	24.2	9,610	86.9	7,038	31.8	26,352	119.2
**Burundi**	2,061	32.4	8,334	1,31.1	1,490	23.8	5,499	87.7	3,551	28.1	13,833	109.6
**Cambodia**	6,155	70.1	23,695	269.8	5,077	60.6	16,939	202.0	11,232	65.4	40,634	236.7
**Cameroon**	6,561	47.0	26,988	193.5	4,389	31.4	16,630	119.1	10,950	39.2	43,618	156.3
**Canada**	117,093	605.8	468,154	2,422.2	127,418	668.5	485,713	2,548.2	244,511	636.9	953,867	2,484.8
**Cape Verde**	153	54.1	587	207.6	143	50.2	470	164.9	296	52.1	1,057	186.2
**Central African Republic**	628	24.8	2,638	104.3	412	16.6	1,619	65.1	1,040	20.7	4,257	84.9
**Chad**	2,031	23.3	7,838	89.9	2,401	27.6	8,417	96.8	4,432	25.5	16,255	93.4
**Chile**	19,959	204.6	77,793	797.5	24,080	253.6	92,089	969.8	44,039	228.8	169,882	882.5
**China**	1,563,417	226.3	5,977,275	865.3	1,542,526	213.9	4,991,699	692.3	3,105,943	220.0	10,968,974	777.0
**Colombia**	43,204	164.7	170,524	650.2	37,232	147.2	133,132	526.5	80,436	156.2	303,656	589.5
**Comoros**	243	54.0	881	195.9	157	34.3	466	101.8	400	44.1	1,347	148.4
**Congo, Democratic People Republic of**	14,270	29.9	57,048	119.7	10,883	22.9	38,797	81.6	25,153	26.4	95,845	100.6
**Congo, Republic of**	933	32.2	3,673	126.6	680	23.5	2,298	79.3	1,613	27.8	5,971	103.0
**Costa Rica**	4,891	188.5	20,019	771.6	4,876	188.4	18,731	723.8	9,767	188.5	38,750	747.7
**Côte d'Ivoire**	6,223	45.2	25,397	184.4	5,012	35.9	18,204	130.3	11,235	40.5	43,601	157.2
**Croatia**	10,075	479.8	39,995	1,904.8	11,474	585.6	43,228	2,206.0	21,549	530.9	83,223	2,050.2
**Cuba**	16,164	283.9	62,362	1,095.1	18,061	321.9	63,675	1,134.8	34,225	302.7	126,037	1,114.8
**Cyprus**	2,599	424.9	10,813	1,767.6	2,369	387.3	8,974	1,467.1	4,968	406.1	19,787	1,617.4
**Czechia**	24,834	455.9	99,180	1,820.8	27,853	526.6	107,841	2,038.7	52,687	490.7	207,021	1,928.2

© 2024 Bizuayehu HM et al. *JAMA Network Open.*

Country or territory	Females				Males				Both sexes			
	2022		2018-2022		2022		2018-2022		2022		2018-2022	
	Survivors[a]	Prevalence[b]	Survivors[c]	Prevalence[d]	Survivors[a]	Prevalence[b]	Survivors[c]	Prevalence[d]	Survivors[a]	Prevalence[b]	Survivors[c]	Prevalence[d]
**Denmark**	19,313	658.0	76,800	2,616.6	21,469	740.3	82,109	2,831.5	40,782	698.9	158,909	2,723.4
**Djibouti**	299	61.8	1,036	214.2	189	35.5	592	111.2	488	48.0	1,628	160.2
**Dominican Republic**	6,603	119.2	25,905	467.7	7,139	129.4	25,408	460.5	13,742	124.3	51,313	464.1
**Ecuador**	11,798	130.3	46,390	512.2	9,072	100.2	32,488	358.7	20,870	115.2	78,878	435.5
**Egypt**	51,532	98.1	202,396	385.3	45,679	85.2	164,427	306.6	97,211	91.6	366,823	345.6
**El Salvador**	3,401	97.5	13,035	373.7	2,426	79.2	8,472	276.6	5,827	89.0	21,507	328.3
**Equatorial Guinea**	336	50.6	1,277	192.3	270	32.4	908	109.1	606	40.5	2,185	146.0
**Eritrea**	671	36.7	2,843	155.7	365	19.9	1,562	85.1	1,036	28.3	4,405	120.3
**Estonia**	3,005	433.1	11,760	1,695.0	3,264	519.7	12,572	2,001.6	6,269	474.2	24,332	1,840.7
**Eswatini**	500	83.2	1,986	330.6	227	38.9	732	125.3	727	61.4	2,718	229.4
**Ethiopia**	26,095	43.2	106,302	176.1	13,471	22.3	52,067	86.1	39,566	32.8	158,369	131.1
**Fiji**	709	157.8	2,854	635.3	466	101.3	1,633	354.8	1,175	129.2	4,487	493.4
**Finland**	15,053	535.0	59,991	2,132.2	16,274	593.7	62,536	2,281.2	31,327	564.0	122,527	2,205.7
**France (metropolitan)**	178,617	527.8	712,901	2,106.5	205,536	647.5	781,913	2,463.3	384,153	585.7	1,494,814	2,279.2
**France, Guadeloupe**	592	274.5	2,297	1,065.1	1,010	548.5	3,974	2,158.1	1,602	400.7	6,271	1,568.5
**France, La Réunion**	943	201.2	3,732	796.3	1,261	287.0	4,665	1,061.7	2,204	242.7	8,397	924.7
**France, Martinique**	692	342.0	2,732	1,350.2	931	542.1	3,713	2,162.1	1,623	433.9	6,445	1,722.9
**French Guyana**	216	136.2	829	522.6	281	180.7	1,044	671.3	497	158.2	1,873	596.2
**French Polynesia**	318	226.5	1,168	832.0	339	235.8	1,045	726.8	657	231.2	2,213	778.8
**Gabon**	796	69.5	3,167	276.4	497	41.9	1,795	151.4	1,293	55.5	4,962	212.8
**Gaza Strip and West Bank**	1,747	66.3	7,092	269.2	1,580	58.3	5,863	216.3	3,327	62.2	12,955	242.4
**Georgia**	4,766	229.3	19,272	927.2	4,999	264.5	18,640	986.1	9,765	246.1	37,912	955.3
**Germany**	233,333	550.7	907,610	2,142.0	262,735	632.9	979,106	2,358.6	496,068	591.4	1,886,716	2,249.2
**Ghana**	9,756	61.1	39,641	248.2	6,621	40.3	24,123	146.9	16,377	50.6	63,764	196.8
**Greece**	22,943	436.8	91,637	1,744.7	26,858	530.3	98,961	1954.1	49,801	482.7	190,598	1,847.5
**Guam**	157	184.5	584	686.3	158	182.8	521	602.8	315	183.6	1,105	644.2
**Guatemala**	6,153	65.3	23,449	248.9	4,525	49.4	15,635	170.7	10,678	57.5	39,084	210.3
**Guinea**	2,772	38.9	11,079	155.3	1,552	23.1	5,313	78.9	4,324	31.2	16,392	118.2
**Guinea-Bissau**	339	32.2	1,447	137.5	183	18.1	740	73.2	522	25.3	2,187	106.0
**Guyana**	440	111.7	1,691	429.2	318	79.5	1,084	271.0	758	95.5	2,775	349.5
**Haiti**	3,374	57.0	12,570	212.5	3,318	57.6	11,041	191.5	6,692	57.3	23,611	202.1
**Honduras**	3,555	69.5	14,464	282.9	2,914	57.0	10,741	210.3	6,469	63.3	25,205	246.6
**Hungary**	24,670	490.5	98,238	1,953.0	25,603	559.5	98,182	2,145.5	50,273	523.3	196,420	2,044.7
**Iceland**	744	433.0	2,916	1,697.0	766	441.3	2,917	1,680.6	1,510	437.2	5,833	1,688.8
**India**	450,712	66.7	1,788,471	264.6	404,772	55.4	1,470,047	201.2	855,484	60.8	3,258,518	231.7
**Indonesia**	150,345	108.5	620,125	447.3	108,847	77.5	397,985	283.3	259,192	92.9	1,018,110	364.7
**Iran, Islamic Republic of**	43,672	102.5	172,531	404.8	50,957	117.4	185,375	427.2	94,629	110.0	357,906	416.1
**Iraq**	14,259	68.5	59,109	283.9	9,750	45.7	37,307	174.8	24,009	56.9	96,416	228.7
**Ireland**	12,042	476.6	48,648	1,925.5	14,786	593.0	58,025	2,326.9	26,828	534.4	106,673	2,124.9
**Israel**	12,873	287.6	52,563	1,174.5	11,593	260.7	45,062	1,013.2	24,466	274.2	97,625	1,094.1
**Italy**	160,213	518.8	634,589	2,054.8	176,760	601.7	659,843	2,246.0	336,973	559.2	1,294,432	2,148.0
**Jamaica**	2,652	176.2	10,541	700.5	2,395	161.8	8,400	567.4	5,047	169.1	18,941	634.5
**Japan**	318,487	495.4	1,221,664	1,900.3	424,291	692.2	1,520,054	2,479.9	742,778	591.5	2,741,718	2,183.2
**Jordan**	4,762	93.7	19,654	386.8	3,772	72.3	14,518	278.1	8,534	82.9	34,172	331.7
**Kazakhstan**	14,873	150.6	61,570	623.3	11,452	122.8	41,275	442.5	26,325	137.1	102,845	535.5
**Kenya**	16,949	59.9	67,630	239.1	9,399	33.7	34,522	123.6	26,348	46.9	102,152	181.7

© 2024 Bizuayehu HM et al. *JAMA Network Open.*

Country or territory	Females				Males				Both sexes			
	2022		2018-2022		2022		2018-2022		2022		2018-2022	
	Survivors[a]	Prevalence[b]	Survivors[c]	Prevalence[d]	Survivors[a]	Prevalence[b]	Survivors[c]	Prevalence[d]	Survivors[a]	Prevalence[b]	Survivors[c]	Prevalence[d]
**Korea, Democratic People Republic of**	18,043	135.9	64,499	485.8	16,206	127.5	51,095	401.9	34,249	131.8	115,594	444.8
**Korea, Republic of**	90,286	352.1	371,277	1,447.8	96,602	376.1	355,652	1,384.6	186,888	364.1	726,929	1,416.2
**Kuwait**	1,787	105.3	7,773	458.0	1,548	57.7	6,116	227.9	3,335	76.1	13,889	317.1
**Kyrgyzstan**	2,556	75.2	10,026	294.9	2,024	60.8	6,857	206.0	4,580	68.1	16,883	250.9
**Lao People's Democratic Republic**	2,738	73.5	10,695	287.0	2,615	69.6	8,788	234.0	5,353	71.6	19,483	260.4
**Latvia**	4,269	428.7	17,010	1,708.3	4,400	515.8	16,941	1,985.9	8,669	468.9	33,951	1,836.4
**Lebanon**	4,487	135.4	17,838	538.1	4,281	127.0	15,738	467.0	8,768	131.2	33,576	502.3
**Lesotho**	712	64.7	2,691	244.5	392	36.5	1,282	119.3	1,104	50.7	3,973	182.6
**Liberia**	1,128	42.8	4,576	173.6	735	27.5	2,634	98.7	1,863	35.1	7,210	135.9
**Libya**	2,824	80.9	11,343	325.0	2,548	71.8	9,293	261.7	5,372	76.3	20,636	293.1
**Lithuania**	5,620	393.0	22,582	1,579.1	7,148	580.4	28,314	2,299.0	12,768	479.7	50,896	1,912.2
**Luxembourg**	1,425	449.2	5,628	1,774.0	1,405	432.2	5,339	1,642.2	2,830	440.6	10,967	1,707.3
**Madagascar**	6,809	46.6	27,968	191.3	4,392	30.2	16,008	110.0	11,201	38.4	43,976	150.7
**Malawi**	6,191	60.6	25,679	251.1	3,737	37.5	13,988	140.5	9,928	49.2	39,667	196.6
**Malaysia**	21,793	135.0	92,326	571.9	17,111	100.4	63,181	370.9	38,904	117.3	155,507	468.7
**Maldives**	189	93.0	718	353.5	182	53.9	603	178.5	371	68.6	1,321	244.2
**Mali**	4,337	40.5	17,445	162.9	2,560	23.8	9,209	85.6	6,897	32.1	26,654	124.1
**Malta**	1,059	478.8	4,143	1,873.0	1,149	515.6	4,216	1,892.0	2,208	497.3	8,359	1,882.5
**Mauritania**	1,142	46.8	4,398	180.4	717	29.1	2,473	100.4	1,859	37.9	6,871	140.2
**Mauritius**	1,235	191.0	5,047	780.4	914	145.6	3,388	539.5	2,149	168.6	8,435	661.7
**Mexico**	81,620	121.5	329,147	489.8	66,664	103.6	248,340	385.8	148,284	112.7	577,487	438.9
**Moldova**	4,894	233.7	19,640	938.0	5,341	278.3	19,665	1,024.6	10,235	255.0	39,305	979.4
**Mongolia**	2,013	117.3	6,580	383.4	2,045	123.1	5,930	356.8	4,058	120.1	12,510	370.3
**Montenegro**	1,023	322.3	4,136	1,303.2	1,019	328.1	3,711	1194.9	2,042	325.2	7,847	1,249.6
**Morocco**	21,997	115.7	89,673	471.7	18,480	98.5	65,211	347.6	40,477	107.2	154,884	410.0
**Mozambique**	7,738	45.5	31,726	186.7	4,779	29.7	17,881	111.1	12,517	37.8	49,607	149.9
**Myanmar**	23,818	83.3	90,968	318.0	19,590	73.6	65,709	246.9	43,408	78.6	156,677	283.7
**Namibia**	1,243	91.6	5,013	369.6	946	74.1	3,590	281.1	2,189	83.1	8,603	326.6
**Nepal**	7,039	43.5	26,615	164.4	5,275	37.6	18,188	129.6	12,314	40.7	44,803	148.2
**New Caledonia**	432	298.2	1,726	1,191.4	494	338.2	1,793	1,227.6	926	318.3	3,519	1,209.6
**New Zealand**	14,302	574.2	58,028	2,329.5	17,034	707.6	65,746	2,731.2	31,336	639.8	123,774	2,526.9
**Nicaragua**	2,892	84.1	11,368	330.6	2,199	65.8	7,866	235.5	5,091	75.1	19,234	283.7
**Niger**	2,703	20.9	10,927	84.3	2,105	16.0	7,418	56.5	4,808	18.4	18,345	70.3
**Nigeria**	42,723	40.0	174,622	163.4	24,920	22.7	94,487	86.0	67,643	31.2	269,109	124.2
**North Macedonia**	2,392	229.9	9,318	895.5	2,834	272.3	9,981	959.0	5,226	251.1	19,299	927.3
**Norway**	16,014	588.2	64,344	2,363.5	18,762	672.7	74,043	2,654.9	34,776	631.0	138,387	2510.9
**Oman**	1,263	69.4	5,445	299.1	1,861	53.1	7,561	215.8	3,124	58.7	13,006	244.3
**Pakistan**	54,117	48.6	216,317	194.2	46,420	39.3	174,126	147.5	100,537	43.8	390,443	170.1
**Panama**	3,159	142.2	12,997	584.8	2,983	134.1	11,673	524.7	6,142	138.1	24,670	554.8
**Papua New Guinea**	3,812	83.8	15,564	342.3	2,880	60.7	10,573	222.8	6,692	72.0	26,137	281.3
**Paraguay**	4,735	131.7	19,132	532.1	4,488	121.0	16,230	437.5	9,223	126.2	35,362	484.0
**Peru**	27,267	160.8	106,114	625.9	22,215	132.8	79,256	473.8	49,482	146.9	185,370	550.3
**Philippines**	71,917	128.3	287,161	512.3	51,072	90.5	178,851	316.8	122,989	109.3	466,012	414.2
**Poland**	77,999	400.9	309,237	1,589.4	82,340	450.4	312,015	1,706.6	160,339	424.9	621,252	1,646.2
**Portugal**	24,469	458.2	98,106	1,837.3	28,037	584.0	105,464	2,196.8	52,506	517.8	203,570	2,007.5

© 2024 Bizuayehu HM et al. *JAMA Network Open.*

Country or territory	Females				Males				Both sexes			
	2022		2018-2022		2022		2018-2022		2022		2018-2022	
	Survivors[a]	Prevalence[b]	Survivors[c]	Prevalence[d]	Survivors[a]	Prevalence[b]	Survivors[c]	Prevalence[d]	Survivors[a]	Prevalence[b]	Survivors[c]	Prevalence[d]
**Puerto Rico**	5,006	336.0	20,540	1,378.7	5,855	436.9	23,203	1731.6	10,861	383.8	43,743	1545.8
**Qatar**	562	74.7	2,348	312.2	879	39.5	3,540	158.9	1,441	48.4	5,888	197.6
**Romania**	35,907	367.2	145,290	1,485.7	41,243	445.8	156,580	1,692.4	77,150	405.4	301,870	1,586.2
**Russian Federation**	256,147	327.5	1,058,705	1,353.7	214,157	316.8	809,560	1,197.6	470,304	322.6	1,868,265	1,281.3
**Rwanda**	2,133	30.9	8,615	124.7	1,771	26.5	6,339	94.8	3,904	28.7	14,954	110.0
**Saint Lucia**	141	150.0	509	541.6	185	203.0	610	669.4	326	176.1	1,119	604.5
**Samoa**	154	157.9	584	598.8	141	134.7	427	407.8	295	145.9	1,011	499.9
**Sao Tome and Principe**	55	48.4	166	146.0	60	52.7	168	147.4	115	50.5	334	146.7
**Saudi Arabia**	11,181	73.8	48,663	321.4	11,445	55.3	46,288	223.6	22,626	63.1	94,951	264.9
**Senegal**	3,976	44.0	15,786	174.9	2,192	25.4	7,726	89.6	6,168	34.9	23,512	133.2
**Serbia**	13,994	316.9	56,074	1,269.8	15,821	373.4	59,060	1,393.9	29,815	344.6	115,134	1,330.6
**Sierra Leone**	1,357	32.6	5,636	135.5	852	20.5	3,244	78.2	2,209	26.6	8,880	106.9
**Singapore**	10,116	357.1	41,956	1,481.1	10,302	331.2	38,587	1,240.4	20,418	343.5	80,543	1,355.1
**Slovakia**	11,212	400.3	45,288	1,616.7	12,807	481.7	49,835	1,874.2	24,019	439.9	95,123	1,742.1
**Slovenia**	5,292	507.6	20,972	2,011.6	6,199	598.7	23,717	2,290.4	11,491	553.0	44,689	2,150.6
**Solomon Islands**	258	72.8	955	269.4	177	48.3	539	147.0	435	60.3	1,494	207.2
**Somalia**	2,130	25.2	8,325	98.6	1,291	15.4	4,658	55.5	3,421	20.3	12,983	77.1
**South Africa**	40,023	129.7	159,612	517.3	33,693	112.7	122,806	410.7	73,716	121.3	282,418	464.8
**South Sudan**	1,767	30.5	7,047	121.5	1,228	21.1	4,500	77.4	2,995	25.8	11,547	99.4
**Spain**	98,039	412.8	398,622	1,678.4	123,551	537.9	474,546	2,066.0	221,590	474.3	873,168	1869.0
**Sri Lanka**	12,685	112.8	50,036	444.8	10,198	98.8	36,472	353.2	22,883	106.1	86,508	401.0
**Sudan**	8,674	37.7	34,571	150.2	5,582	24.3	20,814	90.6	14,256	31.0	55,385	120.4
**Suriname**	398	134.0	1,472	495.5	394	131.5	1,293	431.4	792	132.7	2,765	463.3
**Sweden**	26,755	525.0	106,738	2094.3	32,862	641.5	129,528	2,528.6	59,617	583.4	236,266	2,312.0
**Switzerland**	22,634	512.3	89,594	2,028.0	27,191	624.2	104,980	2,410.1	49,825	567.9	194,574	2,217.7
**Syrian Arab Republic**	6,528	67.4	26,256	271.1	5,427	56.1	19,625	202.7	11,955	61.7	45,881	236.9
**Tajikistan**	2,261	45.7	8,916	180.4	1,765	35.2	6,473	129.1	4,026	40.4	15,389	154.6
**Tanzania, United Republic of**	14,111	44.6	56,390	178.1	9,538	30.1	33,303	105.2	23,649	37.4	89,693	141.7
**Thailand**	65,709	182.4	253,301	703.2	56,703	166.5	188,599	553.8	122,412	174.7	441,900	630.6
**The Netherlands**	53,698	622.2	215,459	2,496.4	57,812	673.8	220,416	2,568.8	111,510	647.9	435,875	2,532.5
**The Republic of the Gambia**	362	28.1	1,406	109.0	277	21.8	884	69.7	639	25.0	2,290	89.5
**Timor-Leste**	302	44.6	1,103	162.8	243	35.1	754	109.0	545	39.8	1,857	135.6
**Togo**	1,637	37.6	6,559	150.5	1,240	28.7	4,524	104.7	2,877	33.1	11,083	127.7
**Trinidad and Tobago**	1,477	207.2	5,917	830.2	1,393	200.8	4,859	700.3	2,870	204.0	10,776	766.1
**Tunisia**	6,352	104.6	25,242	415.7	7,260	121.5	26,106	436.9	13,612	113.0	51,348	426.2
**Türkiye**	81,949	189.1	341,356	787.8	90,982	215.4	337,979	800.3	172,931	202.1	679,335	794.0
**Turkmenistan**	2,571	81.6	10,256	325.7	1,909	62.5	6,807	223.0	4,480	72.2	17,063	275.1
**Uganda**	11,276	46.0	45,918	187.2	8,368	35.0	31,110	130.2	19,644	40.6	77,028	159.0
**Ukraine**	58,077	250.6	233,907	1,009.5	50,480	252.1	180,379	901.0	108,557	251.3	414,286	959.2
**United Arab Emirates**	2,600	82.6	11,511	365.5	2,125	30.7	8,542	123.2	4,725	46.9	20,053	198.9
**United Kingdom**	174,402	503.7	692,387	1,999.8	195,823	578.1	742,935	2,193.2	370,225	540.5	1,435,322	2,095.4
**United States of America**	926,285	547.6	3,762,827	2,224.6	1,051,174	634.5	4,082,871	2,464.6	1,977,459	590.6	7,845,698	2,343.4
**Uruguay**	5,948	329.4	23,500	1,301.6	6,004	355.2	22,509	1,331.5	11,952	341.9	46,009	1,316.0
**Uzbekistan**	13,781	80.0	55,533	322.4	10,239	59.7	37,380	217.9	24,020	69.9	92,913	270.2
**Vanuatu**	103	64.9	375	236.2	77	47.2	224	137.4	180	55.9	599	186.1

© 2024 Bizuayehu HM et al. *JAMA Network Open.*

Country or territory	Females				Males				Both sexes			
	2022		2018-2022		2022		2018-2022		2022		2018-2022	
	Survivors[a]	Prevalence[b]	Survivors[c]	Prevalence[d]	Survivors[a]	Prevalence[b]	Survivors[c]	Prevalence[d]	Survivors[a]	Prevalence[b]	Survivors[c]	Prevalence[d]
**Venezuela**	21,166	142.3	83,755	563.1	19,860	138.0	71,568	497.3	41,026	140.2	155,323	530.7
**Viet Nam**	56,105	113.3	219,715	443.5	56,981	115.3	189,429	383.4	113,086	114.3	409,144	413.5
**Yemen**	4,612	29.8	18,223	117.8	3,525	22.5	13,588	86.6	8,137	26.1	31,811	102.1
**Zambia**	5,008	51.0	20,473	208.3	3,369	34.9	12,549	130.1	8,377	43.0	33,022	169.6
**Zimbabwe**	6,316	78.9	25,178	314.4	3,539	48.3	12,735	173.9	9,855	64.3	37,913	247.3

[a] The number of cancer survivors who diagnosed with cancer in 2022
[b] The one-year (2022) prevalence per 100,000 persons
[c] The number of cancer survivors who diagnosed with cancer over the five-year span (2018-2022)
[d] The five-year (2018-2022) prevalence per 100,000 persons

© 2024 Bizuayehu HM et al. *JAMA Network Open.*

eTable 7. Distribution of Cancer Mortality to Incidence Ratio (MIR), Absolute Differences, and Ratios Compared to the Global MIR in 2022 in Each Country or Territory Sorted by Alphabetical Order

	Females			Males			Both sexes		
**Country or territory**	**MIR**	**Absolute MIR difference[a]**	**Ratio of MIR[b]**	**MIR**	**Absolute MIR difference[a]**	**Ratio of MIR[b]**	**MIR**	**Absolute MIR difference[a]**	**Ratio of MIR[b]**
**Afghanistan**	70.0	28.7	1.7	78.2	27	1.5	74.1	28	1.6
**Albania**	44.9	3.6	1.1	65.9	14	1.3	56.3	10	1.2
**Algeria**	48.0	6.7	1.2	63.3	12	1.2	55.0	8	1.2
**Angola**	66.1	24.8	1.6	69.2	18	1.3	67.4	21	1.4
**Argentina**	42.5	1.2	1.0	53.3	2	1.0	47.6	1	1.0
**Armenia**	53.1	11.8	1.3	62.3	11	1.2	57.9	11	1.2
**Australia**	17.2	-24.1	0.4	19.5	-32	0.4	18.3	-28	0.4
**Austria**	32.5	-8.8	0.8	38.1	-14	0.7	35.3	-11	0.8
**Azerbaijan**	53.9	12.6	1.3	67.8	16	1.3	61.2	15	1.3
**Bahamas**	58.2	16.9	1.4	55.6	4	1.1	56.7	10	1.2
**Bahrain**	45.7	4.4	1.1	56.0	4	1.1	51.5	5	1.1
**Bangladesh**	68.0	26.7	1.6	72.7	21	1.4	70.7	24	1.5
**Barbados**	54.8	13.5	1.3	55.6	4	1.1	55.2	9	1.2
**Belarus**	35.5	-5.8	0.9	53.5	2	1.0	45.2	-1	1.0
**Belgium**	25.0	-16.3	0.6	31.8	-20	0.6	28.5	-18	0.6
**Belize**	48.4	7.1	1.2	56.5	5	1.1	52.2	6	1.1
**Benin**	66.7	25.4	1.6	73.4	22	1.4	69.8	23	1.5
**Bhutan**	72.4	31.1	1.8	80.0	28	1.5	76.7	30	1.6
**Bolivia**	57.2	15.9	1.4	61.3	10	1.2	59.2	13	1.3
**Bosnia Herzegovina**	45.3	4.0	1.1	60.1	8	1.2	53.1	7	1.1
**Botswana**	54.3	13.0	1.3	61.5	10	1.2	57.3	11	1.2
**Brazil**	40.2	-1.1	1.0	44.9	-7	0.9	42.6	-4	0.9
**Brunei Darussalam**	48.2	6.9	1.2	61.5	10	1.2	54.4	8	1.2
**Bulgaria**	43.3	2.0	1.0	58.6	7	1.1	51.4	5	1.1
**Burkina Faso**	77.0	35.7	1.9	81.0	29	1.6	78.7	32	1.7
**Burundi**	76.8	35.5	1.9	77.2	26	1.5	77.0	30	1.7
**Cambodia**	64.2	22.9	1.6	79.7	28	1.5	71.8	25	1.5
**Cameroon**	68.5	27.2	1.7	70.3	19	1.4	69.3	23	1.5
**Canada**	25.6	-15.7	0.6	30.2	-22	0.6	27.9	-19	0.6
**Cape Verde**	62.3	21.0	1.5	69.7	18	1.3	67.0	20	1.4
**Central African Republic**	77.5	36.2	1.9	79.1	27	1.5	78.5	32	1.7
**Chad**	76.0	34.7	1.8	70.8	19	1.4	73.0	26	1.6
**Chile**	47.0	5.7	1.1	48.6	-3	0.9	47.8	1	1.0
**China**	34.4	-6.9	0.8	60.8	9	1.2	47.9	1	1.0
**Colombia**	43.9	2.6	1.1	47.8	-4	0.9	45.8	-1	1.0
**Comoros**	69.8	28.5	1.7	70.9	19	1.4	70.5	24	1.5
**Democratic Republic of Congo**	72.9	31.6	1.8	75.1	23	1.5	74.0	27	1.6

© 2024 Bizuayehu HM et al. *JAMA Network Open.*

Country or territory	Females			Males			Both sexes		
	MIR	Absolute MIR difference[a]	Ratio of MIR[b]	MIR	Absolute MIR difference[a]	Ratio of MIR[b]	MIR	Absolute MIR difference[a]	Ratio of MIR[b]
**Republic of Congo**	61.1	19.8	1.5	70.3	19	1.4	65.6	19	1.4
**Costa Rica**	39.0	-2.3	0.9	45.8	-6	0.9	42.4	-4	0.9
**Côte d’Ivoire**	67.4	26.1	1.6	69.6	18	1.3	68.3	22	1.5
**Croatia**	33.2	-8.1	0.8	46.8	-5	0.9	40.1	-7	0.9
**Cuba**	45.8	4.5	1.1	53.8	2	1.0	49.9	3	1.1
**Cyprus**	30.9	-10.4	0.7	46.5	-5	0.9	38.2	-8	0.8
**Czechia**	32.5	-8.8	0.8	40.9	-11	0.8	37.0	-10	0.8
**Denmark**	26.6	-14.7	0.6	30.4	-21	0.6	28.4	-18	0.6
**Djibouti**	70.2	28.9	1.7	78.6	27	1.5	73.5	27	1.6
**Dominican Republic**	57.0	15.7	1.4	53.8	2	1.0	55.1	9	1.2
**Ecuador**	47.2	5.9	1.1	53.0	1	1.0	49.8	3	1.1
**Egypt**	57.2	15.9	1.4	72.8	21	1.4	64.8	18	1.4
**El Salvador**	50.3	9.0	1.2	51.5	0	1.0	50.9	4	1.1
**Equatorial Guinea**	64.5	23.2	1.6	69.1	17	1.3	67.0	20	1.4
**Eritrea**	70.9	29.6	1.7	75.0	23	1.5	72.7	26	1.6
**Estonia**	34.9	-6.4	0.8	46.2	-6	0.9	40.5	-6	0.9
**Eswatini**	63.9	22.6	1.5	70.4	19	1.4	65.6	19	1.4
**Ethiopia**	69.4	28.1	1.7	74.5	23	1.4	71.3	25	1.5
**Fiji**	58.2	16.9	1.4	53.4	2	1.0	56.4	10	1.2
**Finland**	25.4	-15.9	0.6	32.3	-19	0.6	28.8	-18	0.6
**France (metropolitan)**	27.5	-13.8	0.7	35.1	-17	0.7	31.5	-15	0.7
**France, Guadeloupe**	39.9	-1.4	1.0	35.1	-17	0.7	36.6	-10	0.8
**France, La Réunion**	43.0	1.7	1.0	50.3	-1	1.0	46.9	0	1.0
**France, Martinique**	36.2	-5.1	0.9	35.0	-17	0.7	35.4	-11	0.8
**French Guyana**	38.9	-2.4	0.9	43.8	-8	0.8	40.4	-6	0.9
**French Polynesia**	44.6	3.3	1.1	61.5	10	1.2	53.6	7	1.2
**Gabon**	57.1	15.8	1.4	65.2	14	1.3	60.8	14	1.3
**Gaza Strip and West Bank**	57.2	15.9	1.4	71.0	19	1.4	64.4	18	1.4
**Georgia**	51.1	9.8	1.2	61.5	10	1.2	56.6	10	1.2
**Germany**	33.7	-7.6	0.8	38.7	-13	0.7	36.4	-10	0.8
**Ghana**	64.9	23.6	1.6	73.1	21	1.4	68.6	22	1.5
**Greece**	33.0	-8.3	0.8	44.0	-8	0.9	38.9	-8	0.8
**Guam**	52.0	10.7	1.3	68.0	16	1.3	60.5	14	1.3
**Guatemala**	59.0	17.7	1.4	57.2	6	1.1	58.4	12	1.3
**Guinea**	72.8	31.5	1.8	78.1	26	1.5	75.2	29	1.6
**Guinea-Bissau**	74.8	33.5	1.8	77.7	26	1.5	76.3	30	1.6
**Guyana**	48.6	7.3	1.2	49.7	-2	1.0	49.3	3	1.1
**Haiti**	64.6	23.3	1.6	65.8	14	1.3	65.3	19	1.4
**Honduras**	64.7	23.4	1.6	65.2	14	1.3	65.1	19	1.4
**Hungary**	37.6	-3.7	0.9	47.5	-4	0.9	42.7	-4	0.9
**Iceland**	28.9	-12.4	0.7	33.4	-18	0.6	31.0	-16	0.7
**India**	62.1	20.8	1.5	68.5	17	1.3	65.4	19	1.4

© 2024 Bizuayehu HM et al. *JAMA Network Open.*

	Females			Males			Both sexes		
Country or territory	MIR	Absolute MIR difference[a]	Ratio of MIR[b]	MIR	Absolute MIR difference[a]	Ratio of MIR[b]	MIR	Absolute MIR difference[a]	Ratio of MIR[b]
**Indonesia**	52.2	10.9	1.3	69.3	18	1.3	60.3	14	1.3
**Iran**	63.1	21.8	1.5	65.8	14	1.3	64.8	18	1.4
**Iraq**	55.2	13.9	1.3	69.6	18	1.3	61.8	15	1.3
**Ireland**	29.3	-12.0	0.7	28.2	-24	0.5	28.6	-18	0.6
**Israel**	30.9	-10.4	0.7	37.1	-15	0.7	33.9	-13	0.7
**Italy**	29.9	-11.4	0.7	36.3	-15	0.7	33.1	-14	0.7
**Jamaica**	56.0	14.7	1.4	60.7	9	1.2	58.4	12	1.3
**Japan**	26.2	-15.1	0.6	32.5	-19	0.6	29.4	-17	0.6
**Jordan**	47.9	6.6	1.2	63.0	11	1.2	55.2	9	1.2
**Kazakhstan**	47.0	5.7	1.1	67.2	16	1.3	56.8	10	1.2
**Kenya**	67.1	25.8	1.6	73.9	22	1.4	69.8	23	1.5
**North Korea**	60.6	19.3	1.5	76.4	25	1.5	68.6	22	1.5
**South Korea**	22.3	-19.0	0.5	43.8	-8	0.8	32.8	-14	0.7
**Kuwait**	47.4	6.1	1.1	59.3	8	1.1	54.0	7	1.2
**Kyrgyzstan**	57.8	16.5	1.4	73.9	22	1.4	65.8	19	1.4
**Lao People's Democratic Republic**	62.1	20.8	1.5	79.2	28	1.5	71.1	25	1.5
**Latvia**	35.9	-5.4	0.9	49.0	-3	0.9	42.5	-4	0.9
**Lebanon**	50.4	9.1	1.2	59.2	8	1.1	54.7	8	1.2
**Lesotho**	69.7	28.4	1.7	72.2	21	1.4	71.0	24	1.5
**Liberia**	71.4	30.1	1.7	74.4	23	1.4	72.9	26	1.6
**Libya**	62.0	20.7	1.5	75.3	24	1.5	68.9	22	1.5
**Lithuania**	39.6	-1.7	1.0	46.4	-5	0.9	43.4	-3	0.9
**Luxembourg**	25.8	-15.5	0.6	30.6	-21	0.6	28.3	-18	0.6
**Madagascar**	68.3	27.0	1.7	75.2	24	1.5	71.2	25	1.5
**Malawi**	74.1	32.8	1.8	73.2	22	1.4	73.9	27	1.6
**Malaysia**	54.0	12.7	1.3	67.5	16	1.3	60.7	14	1.3
**Maldives**	43.9	2.6	1.1	64.0	12	1.2	55.8	9	1.2
**Mali**	70.0	28.7	1.7	78.2	27	1.5	73.2	27	1.6
**Malta**	25.7	-15.6	0.6	32.1	-20	0.6	28.9	-18	0.6
**Mauritania**	68.8	27.5	1.7	73.7	22	1.4	71.0	24	1.5
**Mauritius**	41.9	0.6	1.0	55.0	3	1.1	47.6	1	1.0
**Mexico**	43.3	2.0	1.0	47.1	-5	0.9	45.1	-2	1.0
**Moldova**	44.7	3.4	1.1	60.1	8	1.2	52.9	6	1.1
**Mongolia**	72.0	30.7	1.7	81.1	29	1.6	77.0	30	1.7
**Montenegro**	44.7	3.4	1.1	57.2	6	1.1	51.0	4	1.1
**Morocco**	46.9	5.6	1.1	69.6	18	1.3	57.3	11	1.2
**Mozambique**	74.1	32.8	1.8	75.7	24	1.5	74.8	28	1.6
**Myanmar**	65.6	24.3	1.6	78.1	26	1.5	71.7	25	1.5
**Namibia**	56.5	15.2	1.4	60.5	9	1.2	58.4	12	1.3
**Nepal**	65.1	23.8	1.6	71.2	20	1.4	67.8	21	1.5
**New Caledonia**	35.3	-6.0	0.9	43.0	-9	0.8	39.4	-7	0.8

© 2024 Bizuayehu HM et al. *JAMA Network Open.*

Country or territory	Females			Males			Both sexes		
	MIR	Absolute MIR difference[a]	Ratio of MIR[b]	MIR	Absolute MIR difference[a]	Ratio of MIR[b]	MIR	Absolute MIR difference[a]	Ratio of MIR[b]
**New Zealand**	23.3	-18.0	0.6	23.8	-28	0.5	23.5	-23	0.5
**Nicaragua**	54.0	12.7	1.3	61.6	10	1.2	57.5	11	1.2
**Niger**	74.5	33.2	1.8	85.3	34	1.6	79.0	32	1.7
**Nigeria**	61.1	19.8	1.5	72.8	21	1.4	65.7	19	1.4
**North Macedonia**	47.8	6.5	1.2	59.7	8	1.2	54.4	8	1.2
**Norway**	24.7	-16.6	0.6	26.6	-25	0.5	25.7	-21	0.6
**Oman**	53.6	12.3	1.3	67.4	16	1.3	63.0	16	1.4
**Pakistan**	63.3	22.0	1.5	69.2	18	1.3	66.1	20	1.4
**Panama**	40.1	-1.2	1.0	44.9	-7	0.9	42.4	-4	0.9
**Papua New Guinea**	61.8	20.5	1.5	65.3	14	1.3	63.5	17	1.4
**Paraguay**	43.6	2.3	1.1	50.0	-2	1.0	46.8	0	1.0
**Peru**	46.4	5.1	1.1	48.5	-3	0.9	47.4	1	1.0
**Philippines**	53.0	11.7	1.3	70.0	18	1.4	60.9	14	1.3
**Poland**	44.7	3.4	1.1	56.3	5	1.1	50.7	4	1.1
**Portugal**	30.0	-11.3	0.7	43.4	-8	0.8	37.0	-10	0.8
**Puerto Rico**	30.0	-11.3	0.7	36.1	-16	0.7	33.1	-14	0.7
**Qatar**	51.6	10.3	1.2	56.9	5	1.1	56.1	10	1.2
**Romania**	40.4	-0.9	1.0	54.1	2	1.0	47.9	1	1.0
**Russian Federation**	36.8	-4.5	0.9	53.0	1	1.0	44.5	-2	1.0
**Rwanda**	70.0	28.7	1.7	73.1	21	1.4	71.4	25	1.5
**Saint Lucia**	46.4	5.1	1.1	44.9	-7	0.9	45.6	-1	1.0
**Samoa**	46.2	4.9	1.1	59.8	8	1.2	52.6	6	1.1
**Sao Tome and Principe**	66.6	25.3	1.6	63.1	11	1.2	64.8	18	1.4
**Saudi Arabia**	44.8	3.5	1.1	59.8	8	1.2	53.0	6	1.1
**Senegal**	69.4	28.1	1.7	75.0	23	1.5	71.6	25	1.5
**Serbia**	46.4	5.1	1.1	55.5	4	1.1	51.3	5	1.1
**Sierra Leone**	71.2	29.9	1.7	70.5	19	1.4	71.0	24	1.5
**Singapore**	40.2	-1.1	1.0	55.7	4	1.1	47.9	1	1.0
**Slovakia**	40.3	-1.0	1.0	48.1	-4	0.9	44.4	-2	1.0
**Slovenia**	33.6	-7.7	0.8	42.9	-9	0.8	38.3	-8	0.8
**Solomon Islands**	50.8	9.5	1.2	61.7	10	1.2	55.3	9	1.2
**Somalia**	77.2	35.9	1.9	81.9	30	1.6	78.9	32	1.7
**South Africa**	58.1	16.8	1.4	63.0	11	1.2	60.2	14	1.3
**South Sudan**	75.9	34.6	1.8	77.7	26	1.5	76.8	30	1.6
**Spain**	27.4	-13.9	0.7	36.6	-15	0.7	32.3	-14	0.7
**Sri Lanka**	49.6	8.3	1.2	61.3	10	1.2	55.2	9	1.2
**Sudan**	63.5	22.2	1.5	72.8	21	1.4	67.4	21	1.4
**Suriname**	49.2	7.9	1.2	58.9	7	1.1	54.0	7	1.2
**Sweden**	27.7	-13.6	0.7	27.6	-24	0.5	27.5	-19	0.6
**Switzerland**	26.0	-15.3	0.6	28.0	-24	0.5	26.9	-20	0.6
**Syrian Arab Republic**	58.8	17.5	1.4	72.7	21	1.4	65.7	19	1.4
**Tajikistan**	63.4	22.1	1.5	76.3	25	1.5	69.7	23	1.5

© 2024 Bizuayehu HM et al. *JAMA Network Open.*

Country or territory	Females			Males			Both sexes		
	MIR	Absolute MIR difference[a]	Ratio of MIR[b]	MIR	Absolute MIR difference[a]	Ratio of MIR[b]	MIR	Absolute MIR difference[a]	Ratio of MIR[b]
**Tanzania**	66.3	25.0	1.6	73.2	22	1.4	69.3	23	1.5
**Thailand**	51.9	10.6	1.3	69.6	18	1.3	60.5	14	1.3
**The Netherlands**	27.5	-13.8	0.7	33.3	-18	0.6	30.2	-16	0.6
**The Republic of the Gambia**	73.4	32.1	1.8	86.2	35	1.7	79.2	33	1.7
**Timor-Leste**	59.2	17.9	1.4	73.0	21	1.4	65.6	19	1.4
**Togo**	67.0	25.7	1.6	70.9	19	1.4	68.8	22	1.5
**Trinidad and Tobago**	48.9	7.6	1.2	55.8	4	1.1	51.9	5	1.1
**Tunisia**	49.9	8.6	1.2	66.9	15	1.3	59.2	13	1.3
**Türkiye**	39.4	-1.9	1.0	61.5	10	1.2	51.4	5	1.1
**Turkmenistan**	61.6	20.3	1.5	73.5	22	1.4	67.2	21	1.4
**Uganda**	73.2	31.9	1.8	74.5	23	1.4	73.8	27	1.6
**Ukraine**	40.5	-0.8	1.0	61.1	9	1.2	50.6	4	1.1
**United Arab Emirates**	44.6	3.3	1.1	56.4	5	1.1	51.8	5	1.1
**United Kingdom**	30.0	-11.3	0.7	34.0	-18	0.7	31.9	-15	0.7
**United States of America**	21.5	-19.8	0.5	23.2	-29	0.4	22.4	-24	0.5
**Uruguay**	40.1	-1.2	1.0	52.2	1	1.0	45.8	-1	1.0
**Uzbekistan**	58.7	17.4	1.4	68.6	17	1.3	63.4	17	1.4
**Vanuatu**	53.6	12.3	1.3	74.3	23	1.4	62.7	16	1.3
**Venezuela**	47.3	6.0	1.1	51.9	0	1.0	49.4	3	1.1
**Vietnam**	55.0	13.7	1.3	74.9	23	1.4	65.7	19	1.4
**Yemen**	72.3	31.0	1.8	82.0	30	1.6	76.7	30	1.6
**Zambia**	68.6	27.3	1.7	68.1	16	1.3	68.5	22	1.5
**Zimbabwe**	68.4	27.1	1.7	70.3	19	1.4	69.2	23	1.5

[a] The absolute MIR difference between each country/territory and the global values was calculated by subtracting the global MIR from each country/territory value (Absolute Difference in MIR= $MIR_{country/territory} - MIR_{global}$). The negative values are indicated below (lower than) the global values: 41.3%, 51.7% and 46.6% for females, males and both sexes, respectively.

[b] The ratios of MIR between each country/territory and the global value were calculated by dividing the MIR value of each country/territory by the global value (Ratio of MIR= $MIR_{country/territory}$ / MIR_{global}).

© 2024 Bizuayehu HM et al. *JAMA Network Open.*

eFigure 2. Differences in Mortality to Incidence Ratios (MIRs) in Each Country or Territory of 3 Regions Compared With the Global MIR, 2022

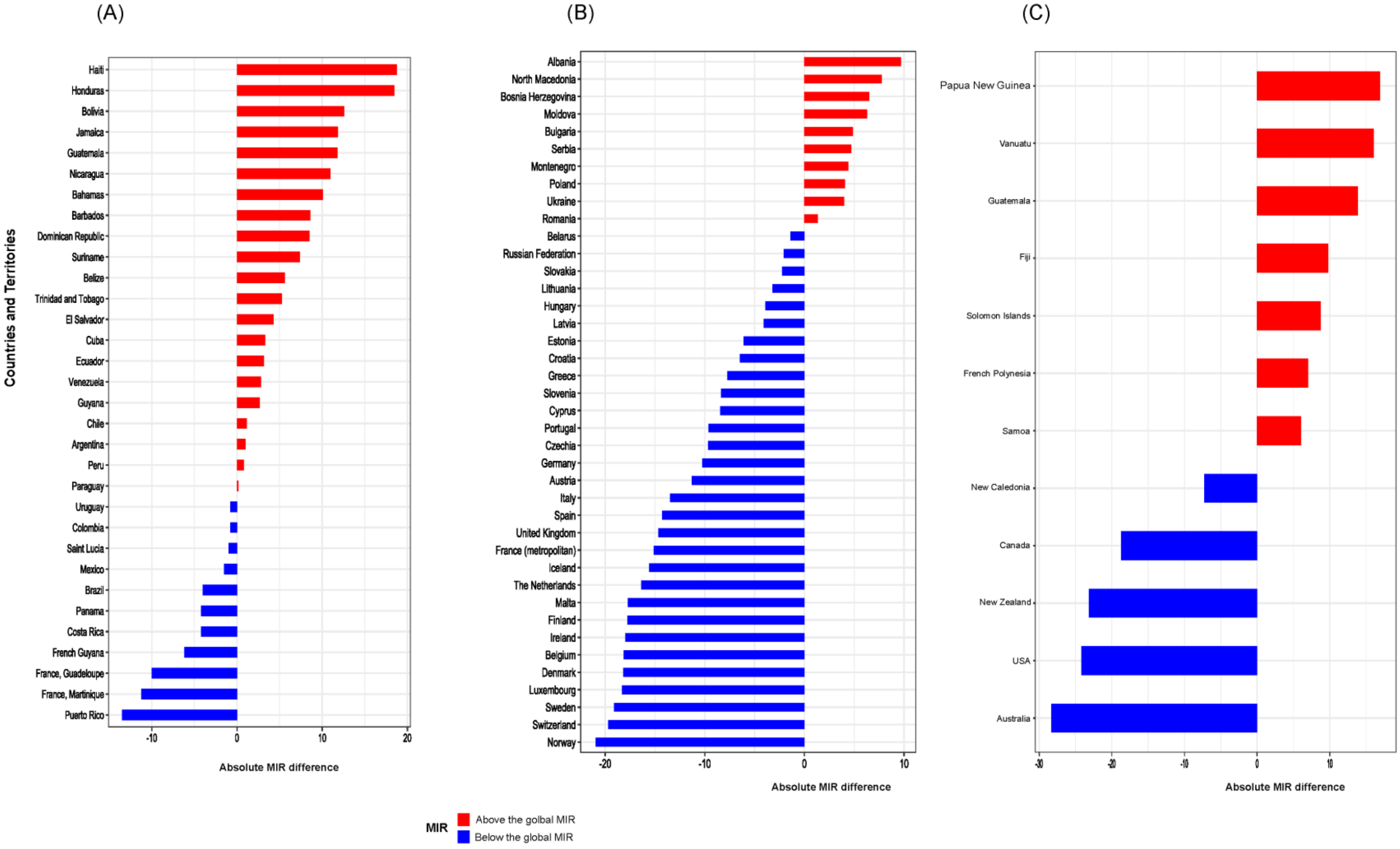

Data are presented for countries in 3 regions: (A) Latin America and the Caribbean, (B) Europe, (C) North America and Oceania. Absolute MIR differences between each country or territory and the global values were calculated by subtracting the global MIR from each country or territory value. The negative values are indicated below (lower than) the global values (46.6%).

© 2024 Bizuayehu HM et al. *JAMA Network Open.*

eReferences

1. Ferlay J, Ervik M, Lam F, Laversanne M, Colombet M, Mery L, Piñeros M, Znaor A, Soerjomataram I, Bray F (2024). Global Cancer Observatory: Cancer Today (version 1.1). Lyon, France: International Agency for Research on Cancer. Available from: https://gco.iarc.who.int/today, accessed [06/04/2024].
2. Ferlay J, Colombet M, Soerjomataram I, et al. Cancer statistics for the year 2020: An overview. *Int J Cancer.* 2021.
3. Sung H, Ferlay J, Siegel RL, et al. Global Cancer Statistics 2020: GLOBOCAN Estimates of Incidence and Mortality Worldwide for 36 Cancers in 185 Countries. *CA Cancer J Clin.* 2021;71(3):209-249.
4. WHO civil registration and vital statistics strategic implementation plan 2021-2025. Geneva: World Health Organization; 2021. Licence: CC BY-NC-SA 3.0 IGO.
5. Sedeta E, Sung H, Laversanne M, Bray F, Jemal A. Recent Mortality Patterns and Time Trends for the Major Cancers in 47 Countries Worldwide. *Cancer Epidemiol Biomarkers Prev.* 2023:OF1-OF12.
6. World Health Organization (2015). International statistical classification of diseases and related health problems, 10th revision, Fifth edition, 2016. World Health Organization. https://apps.who.int/iris/handle/10665/246208.
7. United Nations Development Programme. Human development report. Uncertain Times, Unsettled Lives: Shaping our Future in a Transforming World. 2021-22. cited on 10/03/2024 at: https://hdr.undp.org/content/human-development-report-2021-22.2022.
8. Geifman N, Cohen R, Rubin E. Redefining meaningful age groups in the context of disease. *Age (Dordr).* 2013;35(6):2357-2366.
9. Pedersen JK, Engholm G, Skytthe A, Christensen K, Academy of Geriatric Cancer R. Cancer and aging: Epidemiology and methodological challenges. *Acta Oncol.* 2016;55 Suppl 1(Suppl 1):7-12.
10. Doll R. Cancer incidence in five continents. *A technical Report.* 1966.
11. International Agency for Research on Cancer. Data and methods. Cited on 06/04/2024 at: https://gco.iarc.fr/today/en/data-sources-methods.
12. Asadzadeh Vostakolaei F, Karim-Kos HE, Janssen-Heijnen ML, Visser O, Verbeek AL, Kiemeney LA. The validity of the mortality to incidence ratio as a proxy for site-specific cancer survival. *Eur J Public Health.* 2011;21(5):573-577.
13. Eberth JM, Zahnd WE, Adams SA, Friedman DB, Wheeler SB, Hebert JR. Mortality-to-incidence ratios by US Congressional District: Implications for epidemiologic, dissemination and implementation research, and public health policy. *Prev Med.* 2019;129S:105849.
14. Yang TW, Wang CC, Hung WC, Liu YH, Sung WW, Tsai MC. Improvement in the Mortality-to-Incidence Ratios for Gastric Cancer in Developed Countries With High Health Expenditures. *Front Public Health.* 2021;9:713895.
15. Arnold M, Morgan E, Rumgay H, et al. Current and future burden of breast cancer: Global statistics for 2020 and 2040. *Breast.* 2022;66:15-23.
16. Bizuayehu HM, Dadi AF, Hassen TA, et al. Global burden of 34 cancers among women in 2020 and projections to 2040: Population-based data from 185 countries/territories. *Int J Cancer.* 2023.
17. Wang SC, Sung WW, Kao YL, et al. The gender difference and mortality-to-incidence ratio relate to health care disparities in bladder cancer: National estimates from 33 countries. *Sci Rep.* 2017;7(1):4360.
18. Li J. Digestive cancer incidence and mortality among young adults worldwide in 2020: A population-based study. *World Journal of Gastrointestinal Oncology.* 2022;14(1):278.

© 2024 Bizuayehu HM et al. *JAMA Network Open.*

19. Islami F, Torre LA, Jemal A. Global trends of lung cancer mortality and smoking prevalence. *Transl Lung Cancer Res.* 2015;4(4):327-338.
20. Miranda-Filho A, Pineros M, Bray F. The descriptive epidemiology of lung cancer and tobacco control: a global overview 2018. *Salud Publica Mex.* 2019;61(3):219-229.
21. Choi E, Lee S, Nhung BC, et al. Cancer mortality-to-incidence ratio as an indicator of cancer management outcomes in Organization for Economic Cooperation and Development countries. *Epidemiol Health.* 2017;39:e2017006.
22. R Core Team (2024). R: A Language and Environment for Statistical Computing. R Foundation for Statistical Computing, Vienna, Austria. https://www.R-project.org/.

© 2024 Bizuayehu HM et al. *JAMA Network Open.*
