# Supplementary material for: Global Disparities of Cancer and Its Projected Burden in 2050
Source: JAMA Netw Open. 2024 Nov 5;7(11):e2443198. doi: 10.1001/jamanetworkopen.2024.43198 (PMC11539015; doi:10.1001/jamanetworkopen.2024.43198)
Supplement: Supplement 2. — Data Sharing Statement [file jamanetwopen-e2443198-s002.pdf]

Data Sharing Statement

Bizuayehu. Global Disparities of Cancer and Its Projected Burden in 2050. *JAMA Netw Open*. Published November 05, 2024. doi:10.1001/jamanetworkopen.2024.43198

Data
Data available: Yes
Data types: Deidentified participant data, Other (please specify)
Additional Information: Data can be accessed publicly at https://gco.iarc.fr, with additional information available upon request from the corresponding author.
How to access data: https://gco.iarc.fr
When available: With publication

Supporting Documents
Document types: None

Additional Information
Who can access the data: publicly available for anyone
Types of analyses: for any purpose or for a specified purpose
Mechanisms of data availability: Without investigator support
Any additional restrictions: Additional information available upon request from the corresponding author.
